# CD147-positive migrasome macropinocytosis promotes HCC sorafenib resistance via inducing vasculogenic mimicry triggered by PI3K/AKT/TWIST1 signaling

**DOI:** 10.1038/s41419-026-08972-y

**Published:** 2026-06-15

**Authors:** Luomeng Qian, Ping Chen, Bo Wang, Qing Zhang, Yuan Guo, Jie Cui, Ran Ding, Hongkai Zhang, Sihe Zhang

**Affiliations:** 1 Department of Cell Biology, School of Medicine, Nankai University, Tianjin, China; 2 National Clinical Research Center for Cancer, Key Laboratory of Cancer Prevention and Therapy, Tianjin Medical University Cancer Institute and Hospital, Tianjin, China; 3 Center for AIE Research, Shenzhen Key Laboratory of Polymer Science and Technology, Guangdong Research Center for Interfacial Engineering of Functional Materials, College of Materials Science and Engineering, Shenzhen University, Shenzhen, China; 4 School of Pharmacy, Guangdong Medical University, Dongguan, China; 5 South China University of Technology, Guangzhou, China; 6 State Key Laboratory of Medicinal Chemical Biology, College of Life Sciences, Nankai University, Tianjin, China

**Keywords:** Cancer microenvironment, Membrane trafficking

## Abstract

Sorafenib is a widely used targeting drug for hepatocellular carcinoma (HCC) patients; however, sorafenib resistance poses a significant clinical challenge. Hypoxia and extracellular vesicles (EVs) induce vasculogenic mimicry (VM) which is linked to sorafenib resistance in HCC. Migrasomes (Migs), the newly discovered large EVs, foster HCC malignant phenotypes by facilitating the intercellular transfer of functional molecules. Despite these advancements, the precise mechanism by which Migs cause sorafenib resistance is still unknown. In this study, we found that hypoxia upregulates the Migs release and CD147 expression on Migs surface, which is related to sorafenib resistance in HCC. Further investigations revealed that hypoxia-induced migrasomes (hypo-Migs) induce VM formation, thereby promoting sorafenib resistance in HCC. Notably, hypo-Migs are internalized into HCC cells through macropinocytosis, which depends on the expression of lysophosphatidic acid receptor 6 (LPAR6). Mechanistically, CD147-positive (CD147^+^) hypo-Migs activate the PI3K/AKT/TWIST1 signaling pathway, subsequently inducing VM formation and promoting sorafenib resistance. Importantly, dual blockade of CD147^+^-hypo-Migs macropinocytosis and VM formation enhances the sorafenib-killing efficacy to HCC. Conclusively, our findings uncover a novel sorafenib resistance mechanism induced by CD147^+^-hypo-Migs, and highlight dual-targeting of macropinocytosis and VM as a promising strategy to overcome the sorafenib resistance in HCC.

**Figure:**
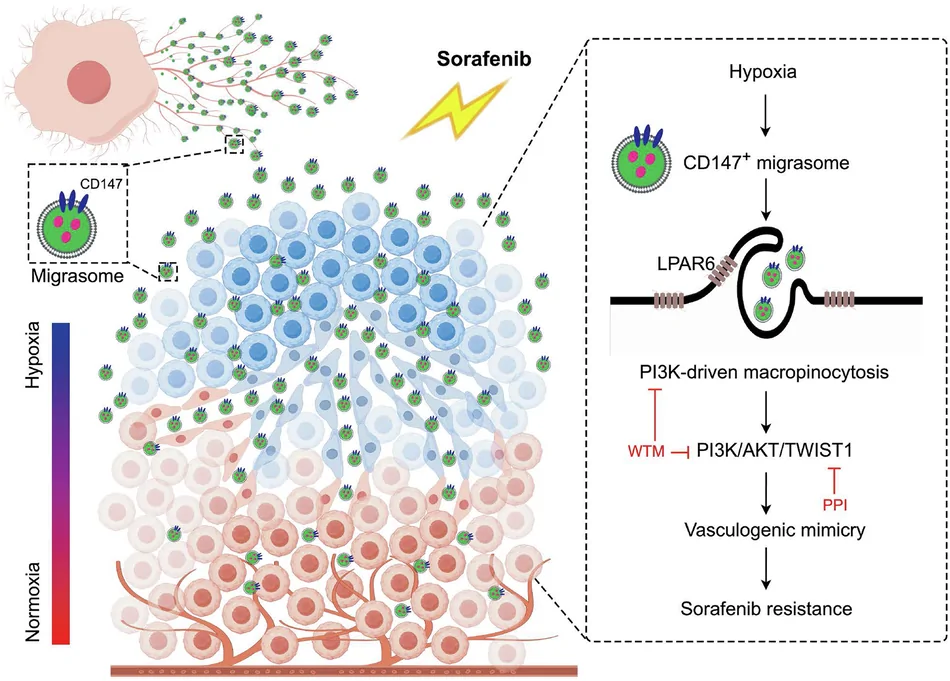

## Introduction

Hepatocellular carcinoma (HCC) is an aggressive malignancy with limited therapeutic options. Sorafenib, a first-line multikinase inhibitor, improves the survival of HCC patients. However, its clinical efficacy is hampered by the development of sorafenib resistance (SFR). A major cause to this resistance is that sorafenib inhibits angiogenesis, leading to a hypoxic microenvironment in HCC [1]. Although hypoxia-caused SFR is recognized [2], the underlying mechanisms have not been fully elucidated.

Hypoxia is a well-established driver that promotes the invasion and metastasis of HCC [3–5]. It also promotes the formation of vasculogenic mimicry (VM) in HCC [6, 7]. VM is an alternative perfusion channel, independent of endothelial cells, formed by malignant cells to provide a blood supply for cancer growth and metastasis. As a vasculogenic-like network in which cancer cells undergo hypoxia and nutrient deficiency, VM is linked to the malignant phenotypes of HCC [8, 9]. Recently, extracellular vesicles (EVs) have been shown to promote VM formation in HCC. Anti-angiogenic therapies induce the release of VEGF-enriched exosomes from endothelial cells. These exosomes promote VM formation and HCC progression [10]. circRNA-100338 is highly expressed in HCC cells and their secreted exosomes. circRNA-100338-enriched exosomes enhance VM formation and favor HCC metastasis [11]. While these studies provide preliminary insight, the contributions of other EV subtypes, especially those secreted from hypoxic and/or metastatic HCC, to VM formation are completely unclear.

Migrasomes (Migs) are newly discovered large EVs that are released from migrating cells [12, 13]. Increasing evidence shows that Migs promote malignancy by orchestrating cell-cell communication, modulating the tumor microenvironment (TME), and facilitating the intercellular transfer of functional molecules [14]. For instance, CD151-enriched Migs promote the invasion and angiogenesis of HCC, thereby facilitating cancer progression [15]. Hypoxia-induced Migs (hypo-Migs) enable colorectal cancer liver metastasis through NRP2/PROX1-mediated education of macrophages toward the efferocytosis phenotype [16]. Cancer-associated fibroblast (CAF)-secreting Migs facilitate HCC progression by boosting the malignant transformation, angiogenesis and immune exclusion [17]. tsRNA-10105-containing Migs exacerbate the HCC immunosuppressive microenvironment by inducing M2 macrophage polarization [18]. Moreover, high expression of Migs-related markers is associated with poor prognosis, metabolic disturbance and immunosuppression in HCC [19–21]. Despite these advances broaden our horizon into Migs-facilitated HCC progression, whether Migs caused SFR remains ambiguous.

In this study, we demonstrated that hypo-Migs induce the VM formation, thereby promoting the SFR in HCC. Hypo-Migs are internalized into HCC cells through macropinocytosis facilitated by lysophosphatidic acid receptor 6 (LPAR6) expression. Specifically, CD147-positive (CD147^+^) hypo-Migs activate the PI3K/AKT/TWIST1 signaling pathway, subsequently inducing VM formation and promoting SFR. Notably, dual blockade of CD147^+^-hypo-Migs macropinocytosis and VM formation substantially enhances the sorafenib-killing efficacy to HCC. Our findings uncover a novel SFR mechanism induced by hypo-Migs and highlight the targeting of macropinocytosis and VM as promising strategies to overcome the SFR in HCC.

## Results

### Hypoxia and increased Migs level are related with the SFR in HCC

**Fig. 1:**
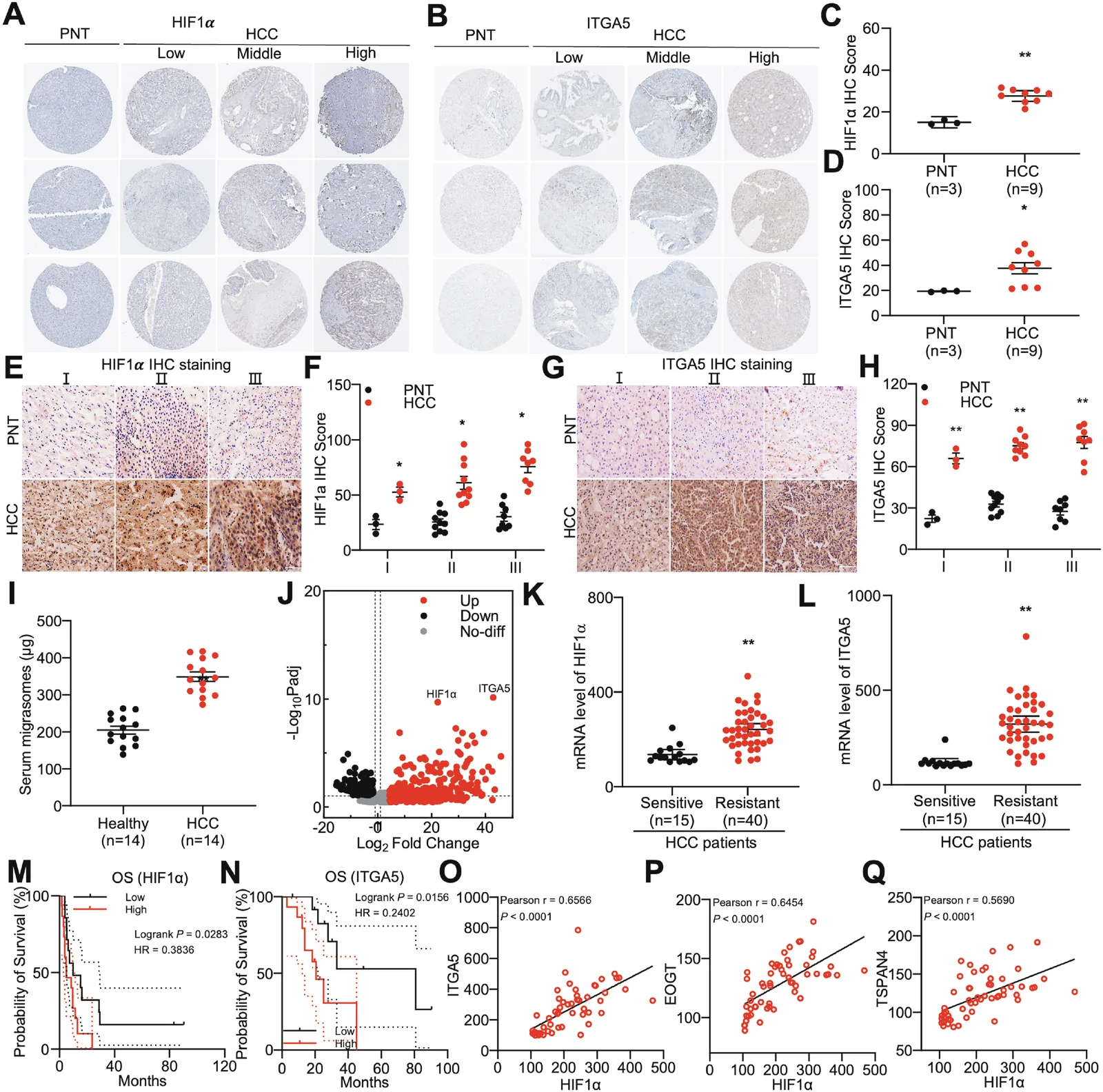
Hypoxia and increased Migs levels are related to the SFR in HCC. IHC staining (**A**, **B**) and quantitative data (**C**, **D**) of HIF1*α* and ITGA5 in PNT and HCC tissue microarrays. Data are derived from HPA database. IHC staining and quantitative data of HIF1*α* (**E**, **F**) and ITGA5 (**G**, **H**) in PNT and HCC tissues at different pathological stages (TNM classification). Scale bars, 100 µm. **I** Serum Migs levels in healthy individuals and HCC patients. **J** mRNA levels of HIF1*α* and ITGA5 in SFR-HCC cells. Data is derived from GEO database (GSE128686). The training dataset was enlarged in equal proportions. mRNA levels of HIF1*α* (**K**) and ITGA5 (**L**) in sorafenib sensitive (SFS)- and SFR-HCC tissues. Data is derived from GEO database (GSE109211). **M**, **N** OS of sorafenib-treated HCC patients with different expression levels of HIF1*α* and ITGA5. Data is derived from TCGA database. **O**–**Q** The expression-correlation (Pearson correlation) analysis of HIF1*α* with ITGA5, EOGT and TSPAN4 in sorafenib-treated HCC tissues. Data are derived from GEO database (GSE109211). All data are presented as means ± SEM. *P < 0.05, **P < 0.01. ns: no significant difference. Student’s *t*-test.

To assess whether hypoxia and Migs levels are increased in HCC, the HPA database was first explored. The typical markers of Migs include integrin *α*5 (ITGA5), EGF domain specific O-linked N-acetylglucosamine transferase (EOGT) and tetraspanin 4 (TSPAN4) [12, 14, 22, 23]. Among these Migs markers, ITGA5 contributes mostly to proliferation, metastasis and SFR of HCC [19, 24], so we first chose ITGA5 as the typical Migs marker for IHC analysis. Tissue microarray analysis showed that hypoxia-inducible factor-1 alpha (HIF1*α*) and ITGA5 were highly expressed in HCC tissues than that in paracancerous tissues (PNT) (Fig. 1A–D). IHC staining further revealed the elevated expression levels of HIF1*α* and ITGA5 in clinically collected HCC tissues (Fig. 1E–H). Notably, HIF1*α* and ITGA5 levels were significantly increased in advanced (TNM classification) HCC tissues (Fig. 1E–H). Meanwhile, serum tests showed that the Migs levels in HCC patients were higher than that in healthy individuals (Fig. 1I). These results indicate that hypoxia and Migs levels are respectively increased in the cancer tissues and serum of HCC patients. To further determine whether hypoxia and Migs levels are associated with SFR, the HIF1*α* and ITGA5 expression levels in SFR-HCC cells and tissues were analyzed. GEO database-mining revealed the higher expression levels of HIF1*α* and ITGA5 in SFR-HCC cells and tissues (Fig. 1J–L). Survival analysis showed that sorafenib-treated HCC patients with high HIF1*α* and ITGA5 expression had short overall survival (OS) (Fig. 1M, N). These results indicate that hypoxia and increased Migs levels are associated with poor prognosis in sorafenib-treated HCC patients. To further investigate whether hypoxia is related to the level of Migs, the expression-correlation between HIF1*α* and Migs markers was analyzed. TCGA database-mining showed that the expression levels of main Migs markers (ITGA5, EOGT and TSPAN4) were positively related with HIF1*α* level (Fig. 1O–Q). Collectively, these clinical and bioinformatics data suggest that hypoxia and high Migs levels are related with the SFR in HCC.

### Hypoxia upregulates Migs release and CD147 expression on Migs surface

**Fig. 2:**
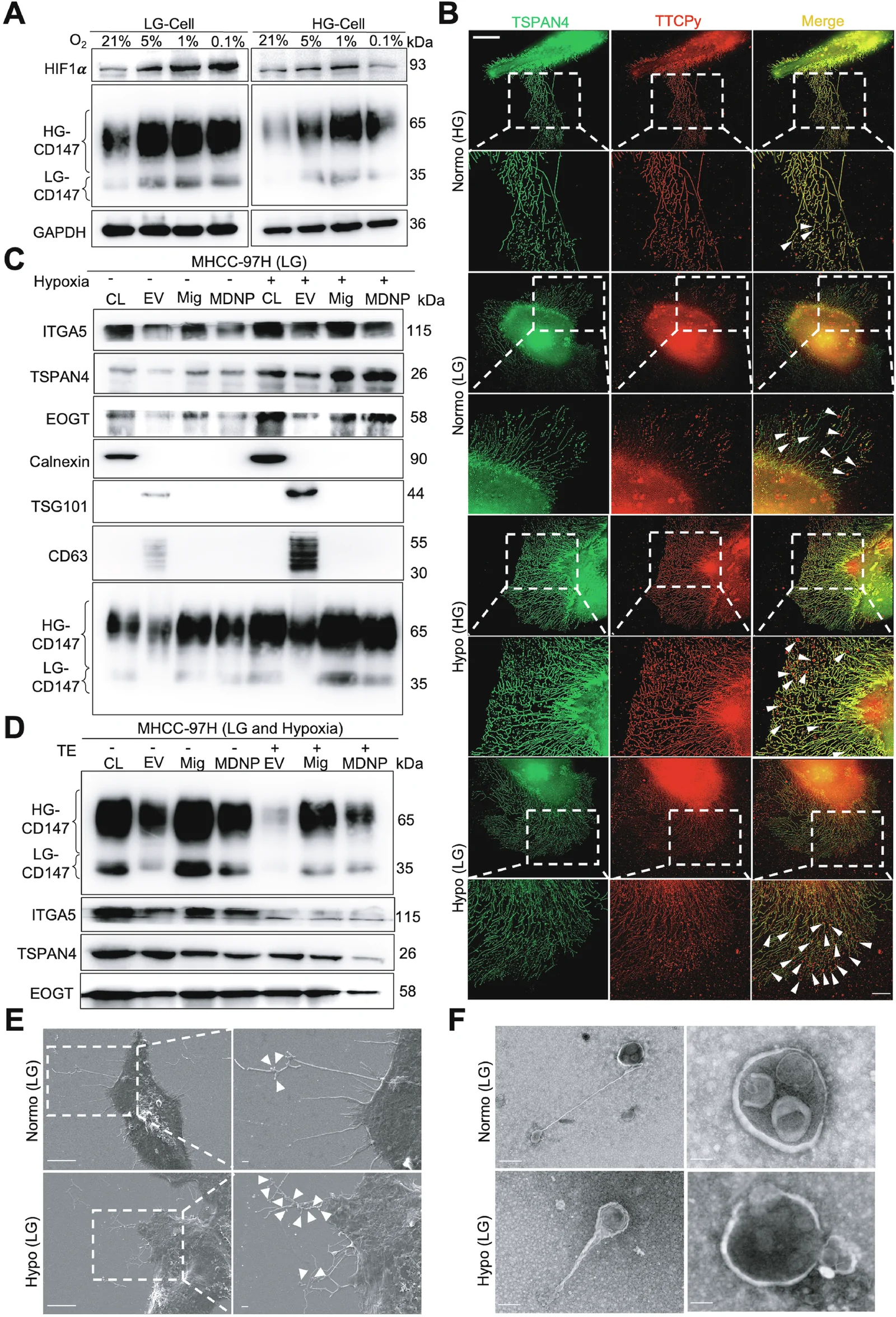
Hypoxia increases the Migs release and CD147 expression on Migs. **A** MHCC-97H cells were cultured under LG- and HG-conditions, and exposed to 21%, 5%, 1%, and 0.1% O_2_. HIF1*α* and CD147 expression levels were determined by Western blotting. **B** HIS-SIM images of hypo- and normo-Migs from MHCC-97H cells cultured under LG- and HG-conditions. Red channel: TTCPy staining (λex = 488 nm, λem = 652–682 nm); Green channel: TSPAN4-GFP (λex = 488 nm, λem = 500–550 nm). Scale bar, 2 μm. **C** Western blotting analysis of the marker expression levels of Migs and EVs isolated from LG-cultured MHCC-97H cells after hypoxia- or normoxia-treatment. Mig-positive markers: ITGA5, TSPAN4 and EOGT. EVs-positive markers: TSG101 and CD63. EVs-negative marker: Calnexin. CL: cell lysate. **D** Western blotting determines the expression levels of CD147 and specific markers (ITGA5, TSPAN4 and EOGT) on hypo-Migs with or without trypsinization. **E** Representative SEM images of MHCC-97H cells cultured under normoxic or hypoxic conditions, with magnified images of Migs on the right. White arrows: Migs. Low magnification: scale bar = 10 µm; high magnification: scale bars = 1 µm. **F** Representative TEM images of isolated hypo- or normo-Migs, with magnified images of Migs on the right. Low magnification: scale bars = 1 µm; high magnification: scale bars = 100 nm.

Being a transmembrane glycoprotein, high expression of CD147 promotes multidrug resistance (MDR) in cancers including HCC [25–28]. To determine whether culture condition influences the CD147 expression level in invasive HCC cells, metastatic MHCC-97H and HCCLM3 cells were low-glucose (LG)- or high-glucose (HG)-cultured under normoxia and hypoxia, respectively. Western blotting showed that LG-culture increased the CD147 expression level in these two cell lines regardless of normoxia and hypoxia. In contrast, hypoxia more markedly increased the CD147 expression level in LG-cultured metastatic HCC cells (Fig. 2A, Supplementary Fig. S1A, B). This phenomenon motivated us to investigate whether hypoxia more markedly increases the level of Migs released from LG-cultured HCC cells. To visualize the Migs clearly, MHCC-97H and HCCLM3 cells were first transfected with TSPAN4-GFP plasmids for exogenous expression, and then labeled with the aggregation-induced emission (AIE) based near-infrared (NIR) probe TTCPy [29]. Since TTCPy staining allows simple, rapid, live-cell super resolution and high-performance imaging of Migs, its colocalization with TSPAN4 (a typical marker highly enriched in Migs) affords markedly improved spatial resolution and signal-to-background ratio. High-sensitivity structured illumination microscopy (HIS-SIM) imaging showed that LG-culture increased the Migs release (white arrows) under normoxic and hypoxic conditions. However, hypoxia more markedly increased the Migs released from LG-cultured MHCC-97H and HCCLM3 cells (Fig. 2B, Supplementary Fig. S2A–C). To validate this trend, Migs, Migs-derived nanoparticles (MDNPs), EVs and cell lysate (CL) were extracted, and the expression of Migs markers was checked. Western blotting showed that hypoxia increased the expression levels of Migs markers (TSPAN4, EOGT, and ITGA5), and EVs-positive markers (TSG101 and CD63) in LG-cultured MHCC-97H and HCCLM3 cells. Moreover, hypoxia also increased the CD147 expression level in these extracts (Fig. 2C, Supplementary Fig. S2G).

To determine whether CD147 is localized on the hypo-Migs surface, the trypsin digestion approach was applied. Western blotting showed that both high-glycosylated CD147 (HG-CD147) and low-glycosylated CD147 (LG-CD147) were present in hypo-Migs, hypoxia-induced EVs (hypo-EVs), hypoxia-induced MDNPs (hypo-MDNPs) and CL extracts. Trypsin digestion sharply reduced the HG-CD147 and LG-CD147 levels on hypo-Migs (Fig. 2D, Supplementary Fig. S2H). These results suggest that CD147 is highly expressed on hypo-Migs from metastatic HCC cells. Further imaging with scanning electron microscopy (SEM) more clearly showed the Migs attaching to cell body via retraction fibers at the edges of MHCC-97H and HCCLM3 cells (Fig. 2E and Supplementary Fig. S2E). Similarly, hypoxia more markedly increased the level of Migs released from LG-cultured metastatic HCC cells (Supplementary Fig. S2B, F). To compare the morphology and size distribution between hypo-Migs and normoxia-induced Migs (normo-Migs), transmission electron microscopy (TEM) and nanoparticle tracking analysis were performed. The results showed that the isolated samples had similar Migs-like structures encapsulating small vesicles with diameters of 0.5–3 µm (Fig. 2F and Supplementary Fig. S3A, B). Collectively, these results suggest that hypoxia increases the Migs released from metastatic HCC cells and CD147 expression on the Migs surface.

### Hypo-Migs promote the SFR by provoking VM formation in HCC

**Fig. 3:**
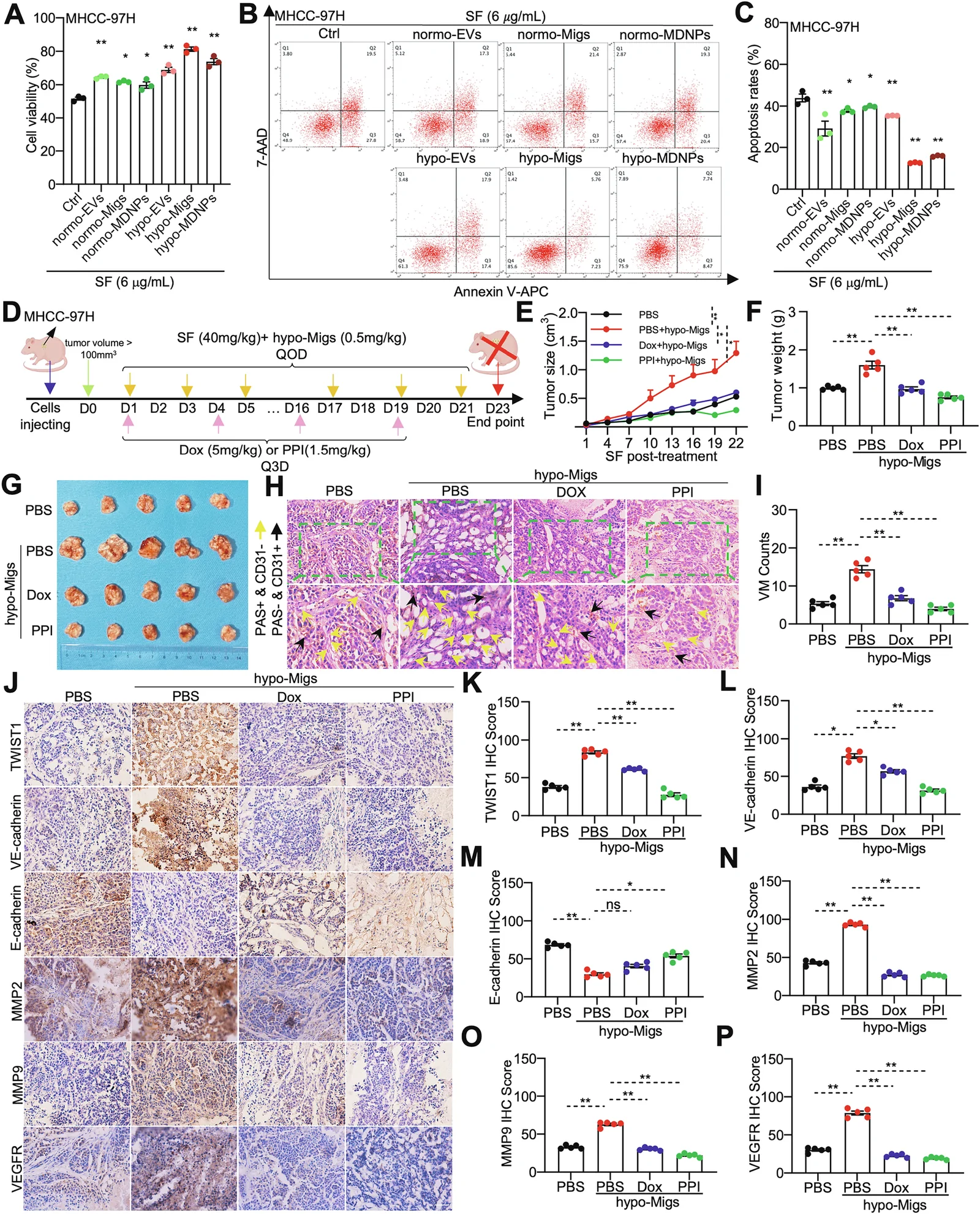
Hypo-Migs promote the SFR by provoking VM formation in HCC. **A** MHCC-97H cells were incubated with/without EVs (normo-EVs, hypo-EVs), Migs (normo-Migs, hypo-Migs) or MDNPs (normo-MDNPs, hypo-MDNPs) (2 μg/well, 24 h) followed by sorafenib treatment (6 μg/mL, 36 h). Cellular viability was determined by CCK8 assay. **B** MHCC-97H cells were preincubated with EVs, Migs, or MDNPs followed by sorafenib treatment. Apoptosis levels were determined via an Annexin V/7-AAD assay, and representative flow cytometry data are shown. **C** Apoptosis variation among treatments is depicted in Box-and-scatter plots. **D** Diagram illustrating the intervention of VM formation in CDX models. HCC-xenografted mice were cotreated with sorafenib (40 mg/kg, i.g., qod, 21 days) and hypo-Migs (500 μg/kg, i.p., qod, 21 days), and further treated with the VM inhibitor DOX (5 mg/kg, i.p., q3d, 21 days) or PPI (1.5 mg/kg, i.p., q3d, 21 days). **E** The volume of HCC tumors was monitored and measured. **F** Weights of excised HCC tissues from different groups. **G** Images of dissected HCC tissues from treated mice. **H** Representative images of CD31- and PAS-staining of xenografted HCC tissues. Yellow arrow: VM; Black arrow: MV. Scale bar: 100 μm. **I** Quantitative data of VM in HCC tissues. **J** Representative IHC staining and **K**–**P** quantitative data of VM-positive markers (TWIST1, VE-cadherin, MMP2, MMP9 and VEGFR) and VM-negative marker (E-cadherin) in xenografted HCC tissues. Scale bars, 100 µm. All the data are presented as means ± SEM. *P < 0.05, **P < 0.01. ns: no significant difference. Student’s *t*-test.

To investigate whether hypo-Migs promote SFR, HCC cells were pretreated respectively with Migs, EVs and MDNPs before sorafenib exposure. CCK8 assays showed that preincubation with hypo-Migs, or hypo-EVs or hypo-MDNPs significantly counteracted the sorafenib-inhibited viability of MHCC-97H and HCCLM3 cells, whereas the normo-counterparts had a relatively weak effect (Fig. 3A and Supplementary Fig. S4A). In parallel, flow cytometry checking showed that normo- and hypo-Migs, EVs and MDNPs both reduced the sorafenib-induced apoptosis, whereas hypo-Migs had the most significant reduction effect (Fig. 3B, C and Supplementary Fig. S4B, C). These results suggest that hypo-Migs most strikingly promote the SFR in HCC cells.

Multiple studies have reported that VM formation promotes the SFR in HCC [8, 24, 30, 31]. To determine whether hypo-Migs promote SFR via inducing VM formation, metastatic HCC cell-derived xenograft (CDX) mouse models were created and co-treated with hypo-Migs and the VM inhibitor doxycycline (DOX) or polyphyllin I (PPI) [6, 32, 33] (Fig. 3D). Tumor volume monitoring showed that, under sorafenib treatment, HCC tumors grew faster in the hypo-Migs group than in the PBS group (Ctrl), indicating that hypo-Migs injection significantly impaired the sorafenib-killing efficacy (Fig. 3E). In contrast, combinational injection with DOX or PPI markedly attenuated HCC growth (Fig. 3E), indicating that hypo-Migs promoted the SFR via inducing VM formation. Endpoint measurements corroborated these findings: hypo-Migs accelerated the xenografted HCC growth, whereas combinational injection with Dox or PPI substantially reduced HCC growth (Fig. 3F, G). Importantly, none of these treatments caused body weight loss (Supplementary Fig. S5A). These results indicate that hypo-Migs promote the SFR, and that VM inhibitors block this promotion in a xenografted HCC model.

Next, VM and microvascular (MV) architectures in HCC tissues were investigated based on CD31 (endothelial cell marker)- and PAS (indicating the basement membrane)-staining. VM was recognized as PAS-positive but CD31-negative staining (PAS^+^/CD31^-^), while MV was identified as PAS-negative but CD31-positive staining (PAS^-^/CD31^+^) (Fig. 3H). The results showed that the VM number in HCC tissues was increased after hypo-Migs injection, whereas combinational injection with DOX or PPI significantly reduced the VM number (Fig. 3H, I). In contrast, no change in MV number was observed after these treatments (Supplementary Fig. S5B). Moreover, IHC staining showed that hypo-Migs markedly increased the expression levels of VM markers (TWIST1, VE-cadherin, MMP2, MMP9, VEGFR) [6, 8, 9, 34], but decreased the expression level of an epithelial marker (E-cadherin) [35] (Fig. 3J–P). In contrast, combinational treatment with DOX or PPI completely reversed the changes in expression levels of hypo-Migs-induced VM and epithelial markers (Fig. 3J–P). Notably, TWIST1 is an EMT regulator highly expressed in cancers that represses E‑cadherin expression but promotes the expression of mesenchymal markers [36]. Thus, these results indicate that hypo-Migs promote SFR by increasing VM formation in HCC.

### LPAR6-dependent macropinocytosis-mediated hypo-Migs uptake promotes the SFR in HCC

**Fig. 4:**
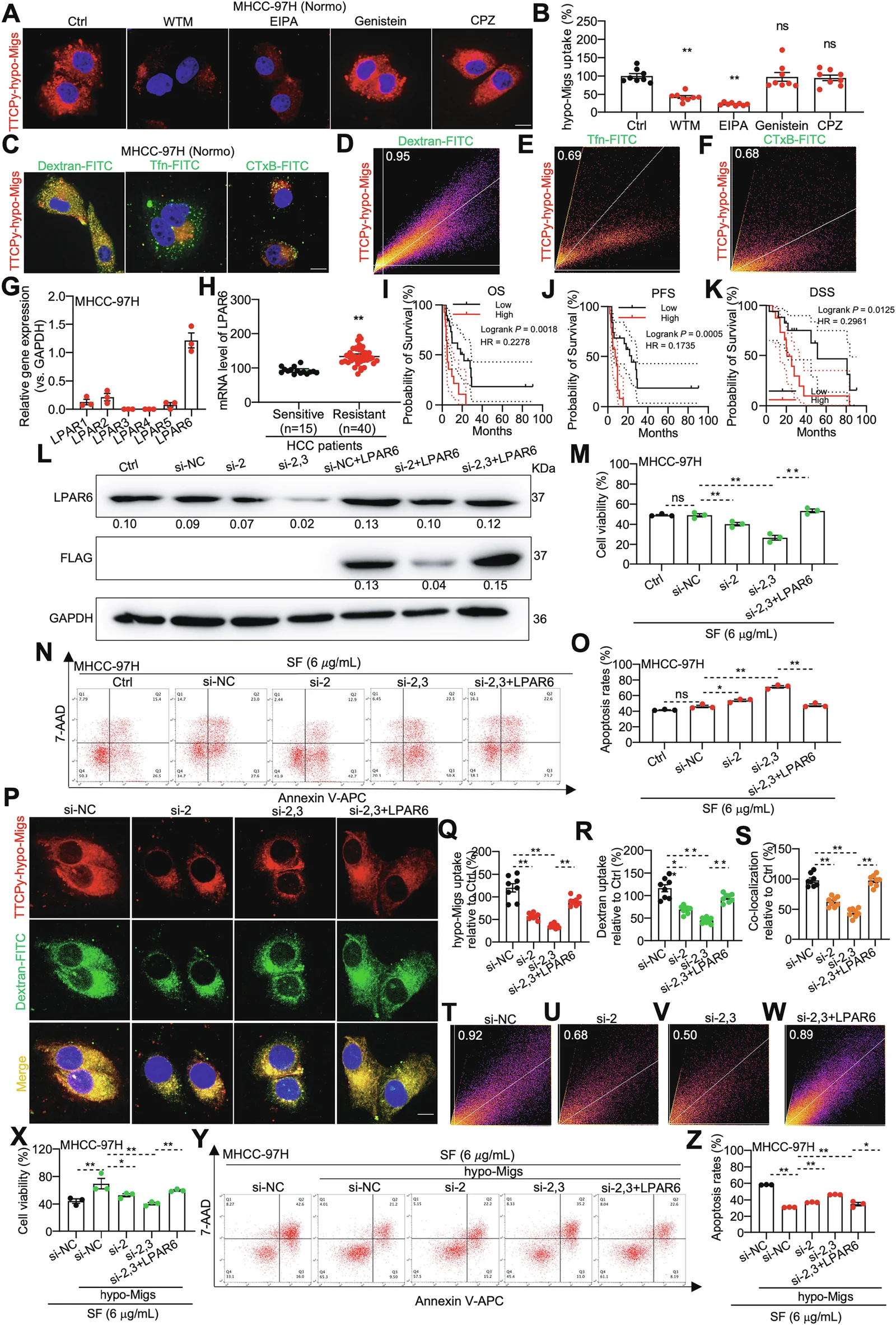
LPAR6-dependent macropinocytosis mediates hypo-Migs uptake, thus promoting the SFR in HCC. **A** Representative confocal images of TTCPy-hypo-Migs uptake (20 μg/mL, 1 h) in MHCC-97H cells pretreated with endocytosis inhibitors for 1 h. WTM (0.5 μM), EIPA (50 μM), Genistein (200 μM) and CPZ (10 μM) were used to inhibit the uptake. Scale bar: 2 μm. **B** Uptake variation of hypo-Migs after different pretreatments is depicted in ox-and-scatter plots. Total particles per cell area were evaluated from at least eight fields. **C** Representative confocal images of the internalized TTCPy-hypo-Migs (1 h, 20 μg/mL) colocalized with FITC-dextran (4 mg/mL), FITC-Tfn (25 μg/mL) or FITC-CTxB (10 μg/mL) in MHCC-97H cells. Scale bar: 2 μm. **D**–**F** Scatterplots illustrating the colocalization between internalized hypo-Migs and endocytosis markers. **G** mRNA levels of LPAR1-6 in MHCC-97H cells were determined by q-PCR. **H** mRNA levels of LPAR6 in SFS- and SFR-HCC tissues. Data is derived from GEO database (GSE109211). **I**–**K** OS, PFS and DSS of sorafenib-treated HCC patients with LPAR6 low- and high-expression. **L** siRNA-mediated LPAR6-KD and LPAR6 rescue in MHCC-97H cells, as determined by Western blotting. **M** MHCC-97H cells with LPAR6-KD and LPAR6 rescue were treated with sorafenib (6 μg/mL, 36 h). Cellular viability was determined by CCK8 assay. **N** Apoptosis levels were determined by an Annexin V/7-AAD assay, and representative flow cytometry data are shown. **O** Apoptosis variation among treatments is depicted by box-and-scatter plots. **P** Representative confocal images of TTCPy-hypo-Migs (20 μg/mL, 1 h) and FITC-dextran (4 mg/mL, 1 h) uptake in MHCC-97H cells with LPAR6-KD and LPAR6 rescue. Scale bar: 2 μm. **Q**, **R** Hypo-Migs and dextran uptake in MHCC-97H cells is depicted in box-and-scatter plots. Total particles per cell were evaluated from at least eight fields. **S** The colocalization coefficient between endocytic hypo-Migs and dextran was analyzed. Total particles per cell area were evaluated from at least eight fields. Ctrl vs si-2, si-2, 3 or si2, 3 + LPAR6. **T**–**W** The co-localization intensity between internalized hypo-Migs and dextran was determined by scatterplots. **X** MHCC-97H cells with LPAR6-KD and LPAR6 rescue were treated with hypo-Migs (2 μg/well, 24 h) and sorafenib (6 μg/mL, 36 h). Cellular viability was determined by CCK8 assay. **Y** Apoptosis level was determined by Annexin V/7-AAD assays, and representative flow cytometry data are shown. **Z** Apoptosis variation among treatments is depicted by ox-and-scatter plots. All the data are presented as means ± SEM. *P < 0.05, **P < 0.01. ns: no significant difference. Student’s *t*-test.

As clathrin-mediated endocytosis (CME), caveolae-dependent endocytosis (CDE) and macropinocytosis are frequent routes for EVs uptake [37], the endocytic route of hypo-Migs in HCC cells was next investigated. Confocal imaging showed that pretreatment with wortmannin (WTM) and 5-(N-ethyl-N-isopropyl) amiloride (EIPA) (both are macropinocytosis inhibitors [38–40]) significantly decreased hypo-Migs uptake in MHCC-97H and HCCLM3 cells. However, pretreatment with genistein (a CDE inhibitor) or chlorpromazine (CPZ; a CME inhibitor) [37, 41, 42] had no effect (Fig. 4A, B and Supplementary Fig. S6A, B). To further determine the endocytic route of hypo-Migs, subcellular colocalization analysis was performed in treated HCC cells. Confocal imaging showed that internalized hypo-Migs colocalized with dextran (a macropinocytosis marker) but minimally colocalized with transferrin (Tfn; a CME marker) or cholera toxin B (CTxB; a CDE marker) [43–45] (Fig. 4C–F and Supplementary Fig. S6C–F). These results indicate that hypo-Migs are taken up by HCC cells via macropinocytosis route.

Lysophosphatidic acid (LPA) stimulates macropinocytosis-mediated EV uptake [28], and contributes to cancer drug resistance by acting on LPA receptors (LPARs) [46, 47]. To investigate whether macropinocytosis-mediated hypo-Mig uptake is dependent on LPARs, their expression profile was examined. Q-PCR analysis showed that LPAR6 is highly expressed in metastatic HCC cells (Fig. 4G and Supplementary Fig. S7A). Therefore, we focused on the role of LPAR6 in hypo-Migs macropinocytosis. First, the colocalization between hypo-Migs and dextran in HCC cells with LPAR6 knockdown (LPAR6-KD) was analyzed (Figs. 4L, P and Supplementary Fig. S8A). Confocal imaging showed that both hypo-Migs and dextran macropinocytosis were reduced in LPAR6-KD MHCC-97H and HCCLM3 cells (Fig. 4Q, R and Supplementary Fig. S8B, C). Further analysis indicated that the colocalization of internalized hypo-Migs with dextran was reduced in LPAR6-KD HCC cells (Fig. 4S–W and Supplementary Fig. S8D–G). In addition, LPAR6 rescue experiments were performed in MHCC-97H cells. Confocal imaging showed that LPAR6 rescue reversed the reduction in hypo-Migs and dextran macropinocytosis in LPAR6-KD cells (Figs. 4L, P–W). These results indicate that macropinocytosis-mediated hypo-Migs uptake is dependent on LPAR6 expression.

To determine whether LPAR6 promotes SFR, its expression profile in HCC tissues was further investigated. GEO database-mining revealed the increased LPAR6 expression in SFR-HCC tissues (Fig. 4H). Survival analysis showed that sorafenib-treated HCC patients with high LPAR6 expression had short OS, progression-free survival (PFS) and disease-specific survival (DSS) (Fig. 4I–K). Importantly, CCK8 assays showed that LPAR6-KD aggravated the sorafenib-inhibited viability in HCC cells (Fig. 4M and Supplementary Fig. S7B). In parallel, flow cytometry analysis showed that LPAR6-KD increased sorafenib-induced apoptosis (Fig. 4N and Supplementary Fig. S7C, D), indicating that LPAR6 expression promotes the SFR in HCC cells. To further investigate whether LPAR6-dependent hypo-Migs macropinocytosis promotes the SFR, CCK8 and flow cytometry analyses were performed again. The results showed that preincubation with hypo-Migs markedly increased sorafenib-inhibited viability, and mitigated sorafenib-induced apoptosis level. Notably, this effect was reversed by LPAR6-KD in MHCC-97H and HCCLM3 cells (Fig. 4X–Z and Supplementary Fig. S9A–C). Similarly, rescue experiment showed that LPAR6 rescue reversed the LPAR6-KD-induced effect on MHCC-97H cells (Figs. 4M–O, X–Z). These results indicate that LPAR6-dependent hypo-Migs macropinocytosis promotes the SFR in metastatic HCC cells.

### CD147^+^-hypo-Migs macropinocytosis promotes SFR via the activation of VM formation-required PI3K/AKT/TWIST1 signaling

**Fig. 5:**
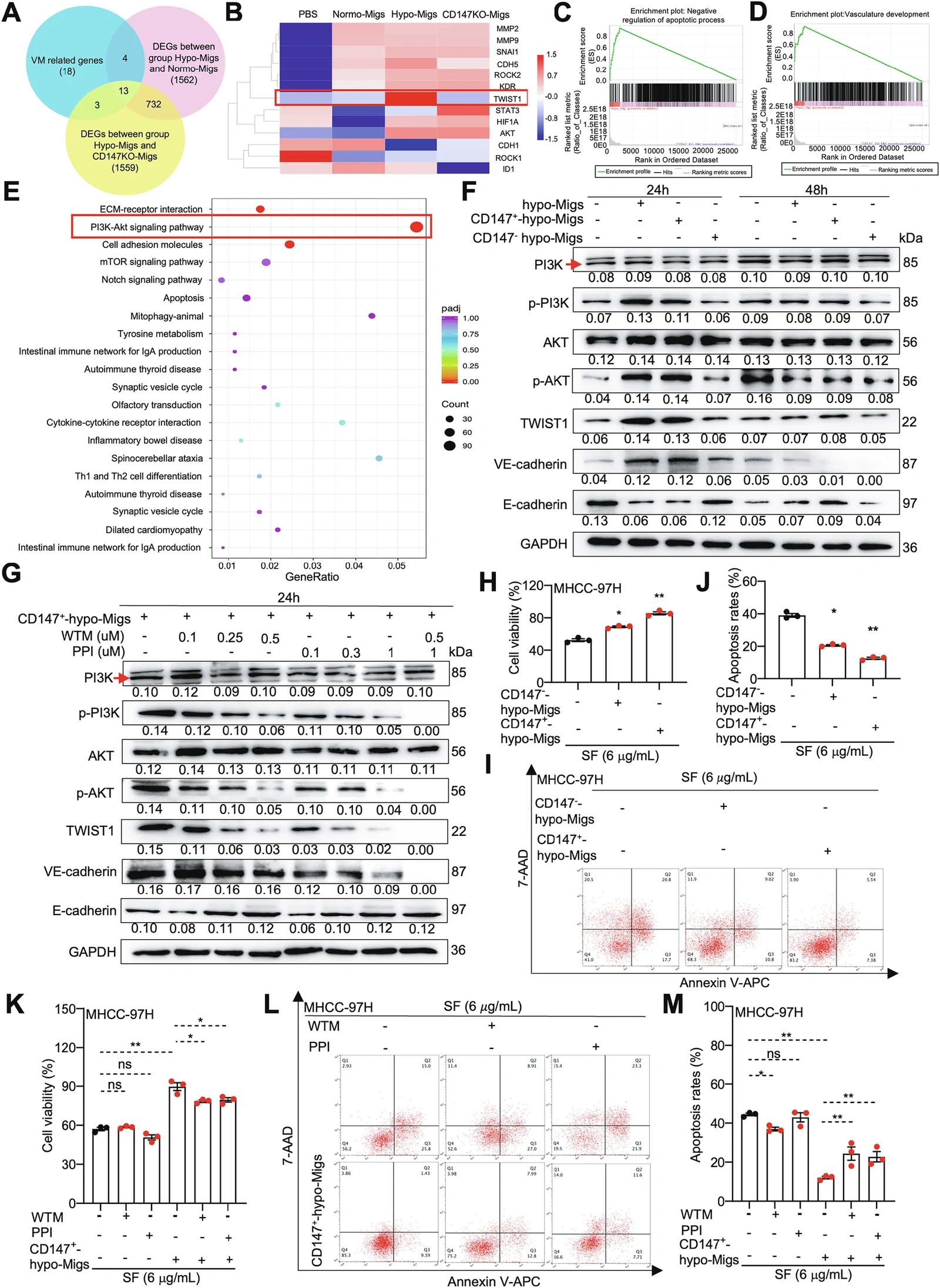
CD147^+^ -hypo-Migs activate PI3K/AKT/TWIST1 signaling to promote the SFR in HCC cells. **A** MHCC-97H cells were treated with normo-Migs, hypo-Migs or CD147KO-Migs. Venn diagram depicting the co-enrichment of VM-related genes with DEGs among hypo-Migs, normo-Migs and CD147KO-Migs groups determined by RNA-seq. **B** Heatmap depicting 13 co-enriched genes from different groups. **C**, **D** GSEA of TWIST1 showing the top two enriched biological pathways in hypo-Migs group. **E** Bubble plot showing the top 20 biological pathways enriched in the KEGG analysis of TWIST1 in hypo-Migs group. Bubble size indicates the gene count, and color depth represents the magnitude of *p*-value. **F** MHCC-97H cells were respectively treated with hypo-Migs, CD147^+^-hypo-Migs and CD147^-^-hypo-Migs (2 μg/well). The p-PI3K, p-AKT, TWIST1, VE-cadherin and E-cadherin levels in MHCC-97H cells were determined by Western blotting. **G** MHCC-97H cells were treated with CD147^+^-hypo-Migs and PI3K inhibitor (WTM) or TWIST1 inhibitor (PPI). Western blot determining the action of inhibitors on Migs-triggered activation of PI3K-AKT-TWIST1-VE-cadherin pathway. **H** MHCC-97H cells were incubated with CD147^+^- or CD147^-^-hypo-Migs (2 μg/well, 24 h) followed by sorafenib treatment (6 μg/mL, 36 h). Cellular viability was determined by CCK8 assay. **I** Apoptosis levels were determined by Annexin V/7-AAD assays, and representative flow cytometry data are shown. **J** Apoptosis variation among treatments is depicted by Box-and-scatter plots. **K**, **L**, **M** MHCC-97H cells were incubated with CD147^+^-hypo-Migs (2 μg/well, 24 h) and WTM or PPI followed by sorafenib treatment (6 μg/mL, 36 h). Cellular viability, apoptosis level and representative data are shown. All the data are presented as means ± SEM. *P < 0.05, **P < 0.01. ns: no significant difference. Student’s *t*-test.

Since CD147 expression promotes VM formation [48], and the aforementioned results show that hypoxia upregulates CD147 expression on Migs, we next determined whether CD147^+^ -hypo-Migs activate VM formation-required signaling. RNA-seq was performed on HCC cells treated with hypo-Migs, normo-Migs, and CD147-knockout (KO, CD147^-^) Migs. Intersection analysis was conducted based on VM-related genes, hypo-Migs-induced differentially expressed genes (DEGs), and CD147^-^Migs-induced DEGs. Venn diagrams showing that 13 VM-related genes (MMP2, MMP9, SNAI1, CDH5, ROCK2, KDR, TWIST1, STAT3, HIF1*α*, AKT, CDH1, ROCK1 and ID1) were significantly enriched [6, 8, 9, 34, 49] (Fig. 5A). Heatmaps showing that hypo-Migs treatment most significantly upregulated TWIST1 expression. In contrast, CD147^-^Migs had no such effect (Fig. 5B). Further Gene Set Enrichment Analysis (GSEA) showed that hypo-Migs treatment mostly enriched two biological pathways: negative regulation of apoptosis and vasculature development (Fig. 5C, D). KEGG analysis showed that PI3K/AKT signaling was mostly associated with TWIST1 function (Fig. 5E). These results suggest that CD147^+^ -hypo-Migs induce VM formation through PI3K/AKT/TWIST1 signaling. To validate this induction, CD147^+^ and CD147^-^-hypo-Migs were respectively cocultured with HCC cells. Western blotting showed that both total Migs and CD147^+^-hypo-Migs treatments increased p-PI3K, p-AKT and TWIST1 levels in HCC cells. Notably, these Migs also upregulated VE-cadherin level but downregulated E-cadherin level (Fig. 5F), indicating the increased VM formation. Such upregulated PI3K/AKT/TWIST1 signaling was still observed when these Migs incubation prolongs to 48 h. In contrast, CD147^-^-hypo-Migs had negligible effects (Fig. 5F). Since WTM inhibits PI3K, which drives macropinocytosis, and PPI impairs VM formation by downregulating TWIST1 expression and inhibiting PI3K/AKT signaling in HCC [6], these two inhibitors were further used. Western blotting showed that both WTM and PPI inhibited the PI3K/AKT/TWIST1 signaling activation and the VM formation induced by CD147^+^-hypo-Migs (Fig. 5G). This inhibition was concentration-dependent and markedly observed when these two inhibitors were combined (Fig. 5G). These results indicate that CD147^+^-hypo-Migs macropinocytosis activates VM formation-required PI3K/AKT/TWIST1 signaling in HCC cells.

To further determine whether CD147^+^-hypo-Migs macropinocytosis promotes SFR via VM formation, CCK8 and flow cytometry assays were carried out again. CCK8 checking showed that CD147^+^-hypo-Migs, but not CD147^-^-hypo-Migs, most significantly counteracted the sorafenib-suppressed viability (Fig. 5H and Supplementary Fig. S10A). In parallel, flow cytometry checking showed that CD147^+^-hypo-Migs significantly mitigated sorafenib-triggered apoptosis (Fig. 5I, J and Supplementary Fig. S10B, C). Furthermore, metastatic HCC cells were pretreated with WTM or PPI followed by CD147^+^-hypo-Migs incubation. CCK8 and flow cytometry checking showed that CD147^+^-hypo-Migs significantly increased the cellular viability but decreased the apoptosis in sorafenib-treated MHCC-97H and HCCLM3 cells. These effects were blocked by WTM- or PPI-pretreatment. No change in viability or apoptosis was observed when these inhibitors were used alone (Fig. 5K–M and Supplementary Fig. S11A–C). These results suggest that CD147^+^-hypo-Migs macropinocytosis promotes SFR via VM formation orchestrated by PI3K/AKT/TWIST1 signaling.

### Dual-targeting CD147^+^-hypo-Migs macropinocytosis and VM formation reverses the SFR of HCC in vivo

**Fig. 6:**
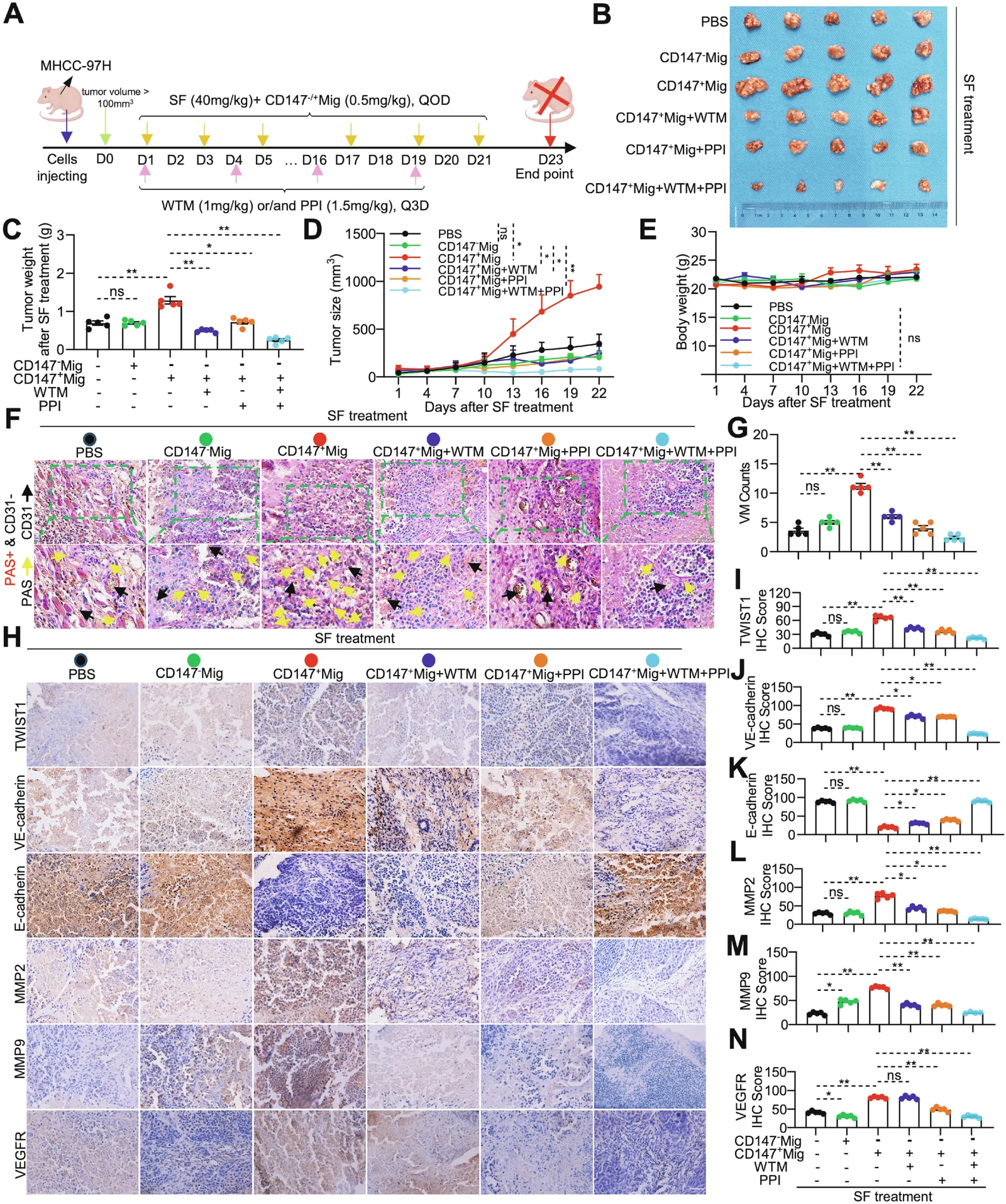
CD147^+^ -hypo-Migs increase the SFR via provoking VM in vivo. **A** Diagram illustrating the therapeutic intervention of VM in CDX models. HCC-xenografted mice were respectively treated with CD147^-^-hypo-Migs, CD147^+^-hypo-Migs, WTM, PPI and sorafenib. **B** Images of dissected HCC tissues from treated mice at day 23 post-administration. **C** Weights of excised HCC tissues from different treatment groups. **D** The volume of HCC tumors was monitored and analyzed every 3 days. **E** The body weights of nude mice were monitored and analyzed. **F** Representative images of CD31- and PAS-staining of HCC tissue sections from treated mice. Yellow arrow, VM; black arrow, MV. Scale bar: 100 μm. **G** Quantitative data of VM in HCC tissue sections from treated mice. **H** IHC staining and **I**–**N** quantitative data of TWIST1, VE-cadherin, E-cadherin, MMP2, MMP9 and VEGFR in HCC tissue sections from treated mice. Scale bars, 100 µm. All data are presented as means ± SEM. *P < 0.05, **P < 0.01. ns: no significant difference. Student’s *t*-test.

To determine whether CD147^+^-hypo-Migs promote SFR via VM formation in vivo, HCC-xenografted mice were treated with CD147^+^- or CD147^+^-hypo-Migs followed by WTM- or PPI-treatment (Fig. 6A). Tumor weight monitoring showed that CD147^+^-hypo-Migs treatment markedly increased HCC growth under sorafenib exposure, whereas WTM- or PPI-cotreatment significantly reversed such an increment. Moreover, further WTM-combined PPT treatment most remarkably reversed the increment (Fig. 6B, C). In contrast, CD147^-^-hypo-Migs treatment did not affect HCC growth (Fig. 6B, C). Tumor volume monitoring showed that, under sorafenib exposure, xenografted HCC tumors grew faster in the CD147^+^-hypo-Migs group than the CD147^-^-hypo-Migs group, indicating that CD147^+^-hypo-Migs impaired sorafenib-killing efficacy (Fig. 6D). In contrast, cotreatment with WTM or PPI substantially slowed HCC growth. Again, further combined treatment exhibited most inhibition on CD147^+^-hypo-Migs-induced HCC growth (Fig. 6D). Additionally, no treatments caused the body weight loss (Fig. 6E). Next, VM and MV architectures in xenografted HCC tissues were analyzed. PAS- and CD31-staining showed that, compared with those in the PBS or CD147^-^-hypo-Migs groups, VM number was markedly increased in CD147^+^-hypo-Migs group. Notably, WTM- or PPI-cotreatment and further WTM-combined PPT treatment both reduced the VM number (Fig. 6F, G). In contrast, CD147^-^-hypo-Migs treatment did not affect MV number (Supplementary Fig. S12). Furthermore, IHC staining showed that CD147^+^-hypo-Migs significantly increased the expression levels of VM markers (TWIST1, VE-cadherin, MMP2, MMP9, and VEGFR) but reduced the expression level of an epithelial marker (E-cadherin) (Fig. 6H–N). In contrast, WTM- or PPI-cotreatment and further WTM-combined PPT treatment significantly reversed these changes (Fig. 6H–N). Together, these results indicate that CD147^+^-hypo-Migs promote SFR via macropinocytosis-mediated VM formation-dependent processes, and that dual-targeting of these functional nodes essentially reverses the SFR of HCC in vivo.

## Discussion

As a hostile TME characteristic, hypoxia favors drug resistance in HCC via complex mechanisms. In this study, HIF1α was highly expressed in SFR-HCC cells and tissues (Fig. 1J, K). This reaffirms that hypoxia favors resistant clone selection under a hypoxic TME in HCC [2]. Beyond this finding, high HIF1*α* expression was correlated with increased migrasome marker expression in HCC (Fig. 1A–H, O–Q), suggesting that hypoxia facilitates the biogenesis and release of Migs. In fact, we found that hypoxia markedly induced the Migs released from HCC cells (Fig. 2B, C, E). This phenomenon is similar to reports that hypoxia promotes EVs secretion in cancers [50–55]. The underlying mechanisms may be: (1) hypoxia facilitates intracellular vesicle trafficking and exocytosis by upregulating Rab GTPases expression [52, 56, 57], (2) hypoxia enhances the expression of tetraspanin and ESCRT-associated proteins to promote EVs release [51, 53, 58–60]. In parallel, we observed that ITGA5, a key marker for Migs formation, is highly expressed in HCC tissues, especially in advanced pathological grade samples (Fig. 1B, D, G, H). High ITGA5 expression is not only correlated with the SFR but also related to poor prognosis in HCC patients (Fig. 1J, L, N). These results align with a report that used ITGA5 as a prognostic and drug (TGX211)-sensitivity marker in HCC [19]. In addition, serum tests showed that the Migs levels in HCC patients were higher than those in healthy individuals (Fig. 1I). On the basis of these results, it’s reasonable to speculate that the serum Migs levels are correlated with the SFR and poor prognosis in HCC patients.

Hypoxia-triggered VM predominantly causes drug resistance in cancers [6, 8, 24, 30, 31]. Despite the fact that both hypoxia and EVs enable VM formation [10, 11, 61, 62], we discovered here that hypo-Migs induce VM formation to promote the SFR in HCC (Fig. 3). Notably, intervention in VM formation by DOX or PPI in CDX models significantly impaired HCC growth (Fig. 3D–P), substantiating that hypo-Migs induce VM formation and the SFR. To our knowledge, this is the first report on Migs-induced VM formation in cancers. Unlike VEGF- or circRNA-enriched exosomes, which facilitates VM formation [10, 11], we found that CD147^+^-hypo-Migs significantly promoted VM formation and the SFR (Fig. 5H–M, Fig. 6). Since CD147 is an inducer of matrix metalloproteinases that enable the forming capillary-like structure [48, 63], CD147^+^-hypo-Migs promoting VM formation is logical. Mechanistically, a key finding here is that CD147⁺-hypo-Migs activate PI3K/AKT/TWIST1 signaling (Fig. 5A–G). As a coenriched VM-related transcription factor (Fig. 5B), TWIST1 not only redirects cancer cell plasticity in VM cells but also increases the drug resistance and metastatic phenotype of HCC [62, 64–66]. Being the mostly associated pathway to TWIST1 function (Fig. 5E), PI3K/AKT signaling was significantly activated by CD147⁺-hypo-Migs (Fig. 5F–G). In fact, activated PI3K/AKT signaling modulates VM formation and drug resistance in HCC-included cancers [67–71]. Therefore, it makes sense in this study that CD147⁺-hypo-Migs activated PI3K/AKT/TWIST1 signaling, thus driving VM formation and SFR (Figs. 5A–G, Fig. 6). This finding also mirrors well to our recent report that CD147⁺ EVs activate AKT/mTOR/4EBP1 signaling to promote SFR [28].

**Fig. 7:**
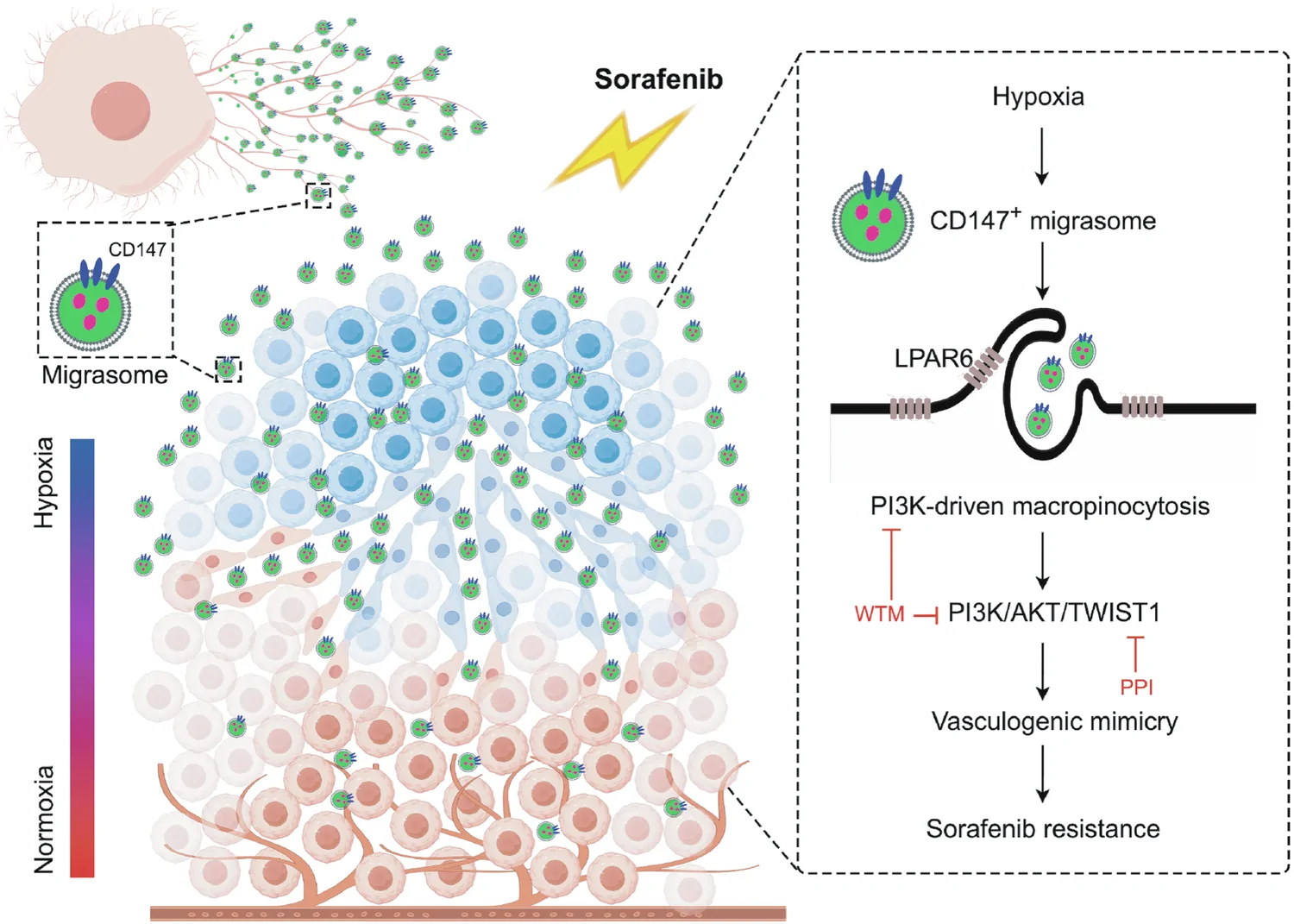
Working model. CD147-positive hypo-Migs promote sorafenib resistance by triggering LPAR6-dependent macropinocytosis and inducing vasculogenic mimicry orchestrated by PI3K/AKT/TWIST1 signaling in HCC.

Macropinocytosis is the bulk endocytosis of extracellular fluid and particles in mammalian cells [38]. In the hostile TME, the uptake of EVs into cancer cells mostly occurs via the macropinocytosis [37, 72–78]. Here, we found that macropinocytosis mediates hypo-Migs uptake into metastatic HCC cells (Fig. 4A–F), thus promoting the SFR (Fig. 5H–M). These findings are consistent with our recent report that LPA-induced macropinocytosis of CD147^+^ EVs in HCC cells [28]. Whether LPA could modulate the hypo-Migs macropinocytosis is a puzzle. Since LPA activates downstream PI3K signaling and the upregulated PI3K drives macropinocytosis in HCC-included cancers [79–81], LPA likely activates PI3K, which subsequently drives CD147^+^ -hypo-Migs macropinocytosis in HCC cells (Fig. 5F–G, Fig. 7). Notably, LPA exerts its biological actions mainly through LPARs, which play indispensable roles in HCC progress [82–84]. Here, we found that LPAR6 was highly expressed in HCC cells and promoted SFR (Fig. 4G–O). Previous studies showed that highly expression of LPAR6 is correlated with poor prognosis in HCC patients [84], and that LPAR6 overexpression promotes the SFR by switching glycolysis to oxidative phosphorylation [85]. Therefore, LPAR6-dependent macropinocytosis clearly mediates hypo-Migs uptake, thus promoting SFR (Fig. 4, Fig. 7).

Despite these discoveries, two limitations remain in this study. First, clinical investigations are currently limited by sample size (Fig. 1E–H), and larger cohorts of sorafenib-treated HCC patients are needed. Second, it is currently difficult to collect peripheral blood from SFR-HCC patients, and correlation analysis between serum-Migs levels and SFR is lack.

In summary, this study reveals a previously unrecognized SFR mechanism orchestrated by CD147-positive hypo-Migs in HCC. Notably, LPAR6-dependent hypo-Migs macropinocytosis activates PI3K/AKT/TWIST1 signaling to induce VM formation and promote the SFR. Importantly, dual blockade of CD147^+^-hypo-Migs macropinocytosis and VM formation substantially enhances the sorafenib-killing efficacy (Fig. 7). Taken together, these data highlight the targeting of macropinocytosis and VM as a novel strategy to overcome SFR in HCC.

## Materials and methods

### Cell culture, plasmids, antibodies, and chemical reagents

Human high metastasis HCC cells, MHCC-97H and HCCLM3, were obtained from the Type Culture Collection of the Chinese Academy of Sciences (China). Cells were routinely cultured in complete DMEM medium with high glucose (HG, 4.5 g/L) and low glucose (LG, 0.45 g/L). All cells were cultured in a humidified atmosphere at 37 °C with 5% CO_2_ in normoxic (21% O_2_) or hypoxic (5%, 1% and 0.1% O_2_) environments. CD147-knockout cell line was supplied by Prof. Zhinan Chen (Air Force Medical University).

FUGW-CAG-TSPAN4-GFP plasmid was supplied by Prof. Yang Chen (Peking University). pCMV-LPAR6 (human)-3×FLAG-Neo (P97737) plasmid was purchased MiaoLingBi.

Rabbit anti-HIF1*α* Ab (WL01607) was purchased from WANLEI BIO. Rabbit anti-CD63 Ab (R23851), anti-TSG101 Ab (R25999), anti-Calnexin Ab (340144), anti-EOGT Ab (R23399), anti-AKT Ab (R23411), anti-VE-cadherin Ab (251821), anti-E-cadherin Ab (340341), anti-CD31 Ab (347526), anti-MMP9 Ab (R380831), anti-VEGFR Ab (R26069), goat anti-mouse Ab-HRP (511103), anti-rabbit Ab-HRP (511203) were purchased from ZENBIO. Goat anti-rabbit Ab-AF594 (SA00013-4), rabbit anti-phospho-AKT Ab (66444-1-lg), and mouse anti-GAPDH Ab (60004-1-Ig) were purchased from Proteintech. Mouse anti-GFP Ab (M20004) was purchased from Abmart. Rabbit anti-LPAR6 Ab was purchased from Immunoway. Rabbit anti-TWIST1 Ab (07769), anti-PI3K Ab (14915), anti-phospho-PI3K Ab (14485), anti-ITGA5 Ab (17098), anti-TSPAN4 Ab (18433) were purchased from UpingBio. Rabbit anti-MMP2 Ab (SRP13502) was purchased from Saier Biotech. Mouse anti-CD147 Ab (H18) was originally produced in our lab [86–88]. Biotin-labeled mouse anti-CD147 Ab (E-AB-F1056B) was purchased from Elabscience.

Streptavidin beads (PuriMag^TM^G) were purchased from PuriMag Biotech. Sorafenib (S45467) was purchased from Yuanye Biotechnology. Doxycycline hydrochloride (D302150) and polyphyllin I (P424367) were purchased from Aladdin. Wortmannin (681675) and Genistein (G810424) were purchased from MACKLIN. EIPA (E10484) was purchased from SAITONG. Chlorpromazine (CPZ, HY-12708) was purchased from MCE. FITC-Dextran (61224ES50) was purchased from YEASEN. FITC-CTxB (C1655) and FITC-Tfn (40-009-090-050) were purchased from SIGMA. Lipofectamine 2000 (Lipo2K, GK20005) was purchased from Good Laboratory Practice Bioscience. Fibronectin (F8180) and CCK8 Kit (CA1210) were purchased from Solarbio. Annexin V/7-AAD apoptosis Kit (A5001-02A-L) was purchased from SIMUBIOTECH. Periodic Acid-Schiff Staining Kit (C0142S) was purchased from Beyotime. An immunohistochemical kit (PV-9000) was purchased from ZSGB-BIO. 100-kDa ultrafiltration tubes (UFC9100) were purchased from Merck Millipore Billerica. The TTCPy molecular probe [29] was supplied by Dr. Jie Cui (Shenzhen University).

### Knock-down of the target gene

Three LPAR6-targeting siRNAs (LPAR6-KD1: GGUUAAUAUCCAAUUGUGU(dT)(dT). LPAR6-KD2: GUUCUUAACCUGUAUUAGU(dT)(dT). LPAR6-KD3: CCUGUUACAUUAAGUAGAA(dT)(dT)) were generated by Tsingke biotech. Lipo2K-based transfection of LPAR6 siRNA duplexes into HCC cells was performed as previously described [28, 87, 89–92].

### Isolation of EVs, migrasomes and migrasome-derived nanoparticles (MDNPs)

The isolation of EVs was performed as our previous study [28] (Supplementary Fig. S13A). The ultrafiltration method was employed to isolate migrasomes and MDNPs as these studies described [23, 93] (Supplementary Fig. S13B).

To isolate CD147⁺ migrasomes, migrasomes were first extracted as described above. The remaining procedure was performed as previously described [28] (Supplementary Fig. S13C).

To isolate migrasomes from serum samples, blood samples of HCC patients with/without sorafenib treatment were collected in pro-coagulation vacuum tubes using standard venipuncture protocols. Large debris was removed by centrifugation at 1000 × *g* for 10 min followed by 4000 × *g* for 20 min. Crude migrasomes were collected by centrifugation at 20,000 × *g* for 30 min, then resuspended with PBS [94].

### Identification of migrasomes

The morphology of isolated migrasomes was checked by transmission electron microscopy (TEM) as the same methods previously described [28, 89, 92, 95].

The size distribution of isolated migrasomes was analyzed by the particle tracking analyzer (Metrix Zetaview nanoparticle). Data was collected over 2 min for each sample.

The protein concentration of the migrasomes and MDNPs’ lysate was determined by the standard BCA protein kit. The expression of migrasomes-specific markers (ITGA5, TSPAN4 and EOGT) was further determined by Western blotting analysis.

### Cellular viability assay

Cellular viability assay was performed by CCK8 kit as previously described [28].

### Cellular apoptosis assay

Cell apoptosis s was measured by the Annexin V/7-AAD apoptosis detection kit according to the manufacturer’s instructions as our previous study [28].

### Migrasomes imaging in HCC cells by HIS-SIM

Migrasomes imaging was measured by high intelligent and sensitive structured illumination microscopy (HIS-SIM, CSR Biotech, Guangzhou) as previously described [29]. Lipo2K-based transient transfection of FUGW-CAG-TSPAN4-GFP plasmid into MHCC-97H cells and HCCLM3 cells was performed and constructed TSPAN4-GFP overexpression cell lines. TTCPy simultaneously labeled migrasomes in TSPAN4-GFP overexpression cell lines and the staining process performed as the research described [29].

### Migrasomes-labeling, uptake and colocalization analysis

Migrasome samples were mixed with TTCPy probe solution in the dark for 30 min. Unlabeled dyes were removed by centrifuging the mixture at 20,000 × *g* for 30 min and labeled migrasome pellet (TTCPy-Migs) was resuspended in PBS.

To determine the migrasomes-uptake pathway, FITC-dextran (70 kDa, macropinocytosis maker, 4 mg/mL), FITC-Tfn (CME marker, 25 μg/mL) and FITC-CTxB (CDE marker, 10 μg/mL) were respectively co-incubated with TTCPy-Migrasomes (20 μg/mL) in HCC cells at 37 °C for 1 h.

Migrasomes uptake measured by confocal imaging was the similar approach as EV uptake [28, 39, 96]. The inhibitor concentrations were EIPA (50 μM), wortmannin (0.5 μM), genistein (200 μM), and CPZ (10 μM). The images were processed by ImageJ software, and the co-localization coefficient was calculated.

### Scanning electron microscope (SEM)

HCC cells were inoculated onto glass slides in 24-well plates at a density of 40%, cultured for 16 h, and fixed overnight with 3% glutaraldehyde at 4 °C. Rinsed the glass slides for 15 min in 0.1 M PBS and dehydrated the glass slides using an ethanol gradient (30%-50%-70%-80%-90%-100%). Lastly, the migrasomes of HCC cells were observed via SEM (JSM-7900F, JEOL, Japan).

### Western blotting

Western blotting was performed as previously described [28, 87, 89–92].

### HCC-xenografted mouse models

HCC cell-derived xenograft (CDX) mouse models were further created as previously described [28, 88, 97]. When the tumor volume reached approximately 0.4 cm in diameter, all mice were randomly grouped (*n* = 5/group) and administered with sorafenib (40 mg kg^−1^, i.g., qod, 21 days) intragastrically every 2 days. Migrasomes (0.5 mg kg^−1^, i.p., qod, 21 days), Doxycycline (5 mg kg^−1^, i.p., q3d, 21 days), Polyphyllin I (2 mg kg^−1^, i.p., q3d, 21 days), and Wortmannin (1 mg kg^−1^, i.p., q3d, 21 days) were intraperitoneally administered twice a week.

### Immunohistochemical staining and PAS/CD31 staining

Peripheral bloods of HCC patients (with/without sorafenib treatment) were collected from the Tianjin Medical University Cancer Institute & Hospital and Tianjin First Central Hospital (China). Immunohistological analysis was performed as described previously [28, 87, 88, 97, 98].

For PAS/CD31 double staining, the procedure was performed as previous described [99, 100]. Sections were stained with CD31 primary antibody (rabbit anti-CD31 Ab, 1:100) and then treated with 0.5% periodic acid solution for 8 min and rinsed with distilled water for 5 min. In a dark chamber, sections were treated with Schiff solution for 30 min at 37 °C.

### Bioinformatics analysis

Bioinformatics analyses were performed similarly as described previously [28, 87, 89, 90, 98]. The Human Protein Atlas (HPA, http://www.proteinatlas.org/) database was employed to analyze the tissue expression level of HIF1*α* and ITGA5. GEO (https://www.ncbi.nlm..nih.gov/gds) database was employed to analyze the mRNA transcriptional level of HIF1*α* and TSPAN4 in HCC tissues (GSE109211) and cell lines (GSE128686). Survival analysis of HIF1*α*, ITGA5 and LPAR6 in HCC patients treated with sorafenib (*n* = 29) was conducted using TCGA database (https://portal.gdc.cancer.gov/). The analysis of gene co-expression between HIF1*α* and ITGA5, EOGT, TSPAN4 was also employed by the TCGA database.

### Transcriptome sequencing (RNA-seq)

Treat MHCC-97H cells with PBS, migrasomes from MHCC-97H cells cultured in hypoxia (2 μg, 24 h, group Normo-Migs), migrasomes from MHCC-97H cells cultured in hypoxia (2 μg, 24 h, group Hypo-Migs) or migrasomes from CD147 knockout MHCC-97H cells cultured in hypoxia (2 μg, 24 h, group CD147KO-Migs). Extract total RNA from these cells and perform subsequent sequencing.

For the differential expression gene (DEG) analysis, DESeq2 provides statistical programs for determining differential expression in digital gene expression data using models based on negative binomial distribution. Padj ≤ 0.05 and |log2(foldchange)| ≥ 1 were used as the screening criteria for DEGs.

The gene set enrichment analysis (GSEA) mainly included three steps: ① According to the expression data of all genes, calculate the difference between groups of each gene (signal2 noise), and then form a well-ordered list of genes. ② Calculate the Enrichment Score (ES) of the gene set. ③ Calculate the significance level of ES.

The KEGG enrichment analysis was implemented by cluster Profiler R package. The corrected *P*-value less than 0.05 were considered significantly enriched. The results were visualized using the online tool (https://magic-plus.novogene.com/#/project/list).

### Statistical analysis

All the results are presented as mean ± SEM values. Statistical analyses were executed with GraphPad Prism (V8.3.0) by using one- or two-way ANOVA or Student’s *t* test. Significant difference was defined as *P < 0.05, **P < 0.01, and ns: no significant difference. The detailed statistical analysis and sample size applied to each experiment was presented in the corresponding figure legends.

### Study approval

Animal experiments were approved by the Nankai University Animal Care and Use Committee (2021-SYDWLL-000479). Human blood samples were obtained from health examiners and HCC patients, and surgical specimens were obtained from HCC patients (informed consent was obtained from the Review Board of Nankai University, NKUIRB2021043). Note: The detailed materials and methods are provided in the Supplementary.

## Supplementary information

Supplementary Figures

Materials and methods (Detailed version)

Full and uncropped western blots

## Data Availability

The data are available upon reasonable request. The RNA-seq data generated in this study have been deposited in the NCBI Gene Expression Omnibus (GEO) database under accession number GSE316455.
