# Supplementary material for: CD147-positive migrasome macropinocytosis promotes HCC sorafenib resistance via inducing vasculogenic mimicry triggered by PI3K/AKT/TWIST1 signaling
Source: Cell Death Dis. 2026 Jun 15;17(1):750. doi: 10.1038/s41419-026-08972-y (PMC13500725; doi:10.1038/s41419-026-08972-y)
Supplement: Supplementary file 1 [file 41419_2026_8972_MOESM1_ESM.docx]

**Fig. S1 LG- and hypoxia-culture upregulates the CD147 expression level.**

**(A)** HCCLM3 and **(B)** Huh7 cells were treated with different concentrations of O_2_, and HIF1𝛼 and CD147 expression levels were determined by Western blotting.

**Fig. S2 Hypoxia culture increases the release of migrasomes from metastatic HCC cells.**

**(A**) Number of hypo- and normo-Migs in MHCC-97H cells cultured under LG- or HG-condition. Images were acquired by HIS-SIM. **(B**) Number of hypo- and normo-Migs in MHCC-97H cells cultured under LG-condition. Images were acquired by SEM. **(C)** Representative super-resolution images of hypo- and normo-Migs in HCCLM3 cells cultured under LG- or HG-condition. Images were acquired by HIS-SIM. Red channel: TTCPy (2𝜇g/mL, 15min. λex = 488 nm, λem = 652-682 nm); Green channel: TSPAN4-GFP (λex = 488 nm, λem = 500-550 nm). Scale bar, 2 μm. **(D)** Number of hypo- and normo-Migs in HCCLM3 cells cultured under LG- or HG-condition. Images were acquired by HIS-SIM. **(E)** Representative SEM images of HCCLM3 cells cultured under normoxia or hypoxia condition, with magnified images of migrasomes on the right. White arrows point to migrasomes. Low magnification: scale bars = 2 µm; high magnification: scale bars = 0.2 µm. **(F)** Number of hypo- and normo-Migs in MHCC-97H cells cultured under LG condition. Images were acquired by SEM. **(G)** Western blotting determined the marker expression levels of Migs and EVs isolated from LG-cultured HCCLM3 cells after hypoxia- or normoxia-treatment. Mig-positive markers: ITGA5, TSPAN4 and EOGT. EV-positive markers: TSG101 and CD63. EV-negative marker: Calnexin. CL: cell lysate. **(H)** Western blotting determined the expression levels of CD147 and specific markers (ITGA5, TSPAN4 and EOGT) on hypo-Mig with or without trypsinization in HCCLM3 cells. All data are presented as means ± SEM. * *P* < 0.05, ** *P* < 0.01. ns: no significant difference. Student’s *t*-test.

**Fig. S3 Hypoxia culture does not change the morphology of migrasomes.**

**(A**, **B**) Size distribution of the isolated normo- and hypo-Migs analyzed by Zetasizer Nano.

**Fig. S4 Hypoxia-induced migrasomes promote the sorafenib resistance of HCC cells.**

**(A)** HCCLM3 cells were incubated with/without hypo- or normo-EVs, Migs or MDNPs (2 μg/well, 24 h) followed by sorafenib treatment (8 μg/mL, 36 h). Cellular viability was determined by CCK8 assay. **(B)** Apoptosis levels of HCCLM3 cells treated with hypo- and normo-EVs, or Migs or MDNPs and sorafenib were determined by Annexin V/7-AAD assay, and representative flow cytometry data are shown. **(C)** Apoptosis variation among treatments is depicted in Box-and-scatter plots. All data are presented as means ± SEM. * *P* < 0.05, ** *P* < 0.01. ns: no significant difference. Student’s *t*-test.

**Fig. S5 Treatment with hypo-Migs and VM inhibitors do not affect the MV counts and body weights of nude mice.**

**(A)** The body weight of nude mice was monitored and analyzed. **(B)** Quantitative data of MV in the HCC tissue sections. All data are presented as means ± SEM. * *P* < 0.05, ** *P* < 0.01. ns: no significant difference. Student’s *t*-test.

**Fig. S6 Normoxic HCC cells intake hypo-Migs via the macropinocytosis pathway.**

**(A)** Confocal images of TTCPy-hypo-Migs uptake (20 μg/mL, 1 h) in HCCLM3 cells. Different endocytosis inhibitors, WTM (0.5 μM), EIPA (50 μM), Genistein (200 μM) and CPZ (10 μM), were used to pretreat cells (1 h) for inhibiting the hypo-Migs uptake. Scale bar: 2 μm. **(B)** Hypo-Migs uptake after different pretreatments is depicted in Box-and-scatter plots. Total particles per cell area were evaluated from at least eight fields. **(C)** Confocal images of the uptake (1 h) of TTCPy-hypo-Migs (20 μg/mL) colocalized with FITC-dextran (4 mg/mL), FITC-Tfn (25 μg/mL) or FITC-CTxB (10 μg/mL) in HCCLM3 cells. Scale bar: 2 μm. **(D-F)** Co-localization coefficient between the internalized hypo-Migs and endocytosis markers is determined. All data are presented as means ± SEM. * *P* < 0.05, ** *P* < 0.01. ns: no significant difference. Student’s *t*-test.

**Fig. S7 Highly expression of LPAR6 promotes the sorafenib resistance to HCC cells.**

**(A)** mRNA levels of LPAR1-6 in HCCLM3 cells were determined by q-PCR. **(B)** HCCLM3 cells with/without LPAR6 knock-down were treated with sorafenib (8 μg/mL, 36 h). Cellular viability was determined by CCK8 assay. **(C)** Apoptosis levels were determined by Annexin V/7-AAD assay, and representative flow cytometry data are shown. **(D)** Apoptosis variation among treatments is depicted in Box-and-scatter plots. All data are presented as means ± SEM. * *P* < 0.05, ** *P* < 0.01. ns: no significant difference. Student’s *t*-test.

**Fig. S8 LPAR6-expression promotes the macropinocytosis of hypo-Migs in HCCLM3 cells.**

**(A)** Confocal imaging the uptake of TTCPy-hypo-Migs (20 μg/mL, 1 h) and FITC-dextran (4 mg/mL, 1 h) in LPAR6-KD HCCLM3 cells. Scale bar: 2 μm. **(B**, **C)** The uptake of hypo-Migs and dextran in HCCLM3 cells is depicted in Box-and-scatter plots. Total particles per cell area were evaluated from at least eight fields. **(D)** co-localization coefficient between endocytic hypo-Migs and dextran is analyzed. Ctrl vs si-2 or si-2,3. **(E-G)** Co-localization intensity between internalized hypo-Migs and dextran is determined by scatterplots. All data are presented as means ± SEM. * *P* < 0.05, ** *P* < 0.01. ns: no significant difference. Student’s *t*-test.

**Fig. S9 Normoxia HCC cells intake hypo-Migs to promote the sorafenib resistance via LPAR6-dependent macropinocytosis.**

**(A)** HCCLM3 cells with/without LPAR6 knock-down were incubated with hypo-Migs (2 μg/well, 24 h) followed by sorafenib treatment (8 μg/mL, 36 h). Cellular viability was determined by CCK8 assay. **(B)** Apoptosis level was determined by Annexin V/7-AAD assay, and representative flow cytometry data are shown. **(C)** Apoptosis variation among treatments is depicted by Box-and-scatter plots. All data are presented as means ± SEM. * *P* < 0.05, ** *P* < 0.01. ns: no significant difference. Student’s *t*-test.

**Fig. S10 CD147-positive hypo-Migs promote the sorafenib resistance in HCCLM3 cells.**

**(A)** HCCLM3 cells were incubated with CD147^+^ or CD147^-^-hypo-Migs (2 μg/well, 24 h) followed by sorafenib treatment (8 μg/mL, 36 h). Cellular viability was determined by CCK8 assay. **(B)** Apoptosis level was determined by Annexin V/7-AAD assay, and representative flow cytometry data are shown. **(C)** Apoptosis variation among treatments is depicted by Box-and-scatter plots. All data are presented as means ± SEM. * *P* < 0.05, ** *P* < 0.01. ns: no significant difference. Student’s *t*-test.

**Fig. S11 CD147-positive hypo-Migs activate PI3K/AKT/TWIST1 signaling to increase the sorafenib resistance in HCC cells.**

**(A)** HCCLM3 cells were incubated with CD147^+^-hypo-Migs (2 μg/well, 24 h) and WTM or PPI followed by sorafenib treatment (8 μg/mL, 36 h). Cellular viability was determined by CCK8 assay. **(B)** Apoptosis level was determined by Annexin V/7-AAD assay, and representative flow cytometry data are shown. **(C)** Apoptosis variation among treatments is depicted by Box-and-scatter plots. All data are presented as means ± SEM. * *P* < 0.05, ** *P* < 0.01. ns: no significant difference. Student’s *t*-test.

**Fig. S12** Quantitative data of MV in HCC tissue sections. All data are presented as means ± SEM. * *P* < 0.05, ** *P* < 0.01. ns: no significant difference. Student’s *t*-test.

**Fig. S13** Isolation of EVs, migrasomes and MDNPs.
