# Supplementary material for: CD147-positive migrasome macropinocytosis promotes HCC sorafenib resistance via inducing vasculogenic mimicry triggered by PI3K/AKT/TWIST1 signaling
Source: Cell Death Dis. 2026 Jun 15;17(1):750. doi: 10.1038/s41419-026-08972-y (PMC13500725; doi:10.1038/s41419-026-08972-y)
Supplement: Supplementary file 2 [file 41419_2026_8972_MOESM2_ESM.docx]

**Materials and methods**

**1. Cell culture, plasmids, antibodies, and chemical reagents.**

Human high metastasis HCC cells, MHCC-97H and HCCLM3, were obtained from the Type Culture Collection of the Chinese Academy of Sciences (China. STR profiling is duly qualified). Cells were routinely cultured in complete DMEM medium with high glucose (HG, 4.5g/L) and low glucose (LG, 0.45g/L). All cells were cultured in a humidified atmosphere at 37 °C with 5% CO_2_ in normoxic (21% O_2_) or hypoxic (5%, 1% and 0.1% O_2_) environments and ruled out the mycoplasma contamination. CD147-knockout cell line was supplied by Prof. Zhinan Chen (Air Force Medical University).

FUGW-CAG-TSPAN4-GFP plasmid was supplied by Prof. Yang Chen (Peking University). pCMV-LPAR6(human)-3×FLAG-Neo (P97737) plasmid was purchased MiaoLingBi.

Rabbit anti-HIF1𝛼 Ab (WL01607) was purchased from WANLEI BIO. Rabbit anti-CD63 Ab (R23851), anti-TSG101 Ab (R25999), anti-Calnexin Ab (340144), anti-EOGT Ab (R23399), anti-AKT Ab (R23411), anti-VE-cadherin Ab (251821), anti-E-cadherin Ab (340341), anti-CD31 Ab (347526), anti-MMP9 Ab (R380831), anti-VEGFR Ab (R26069), goat anti-mouse Ab-HRP (511103), anti-rabbit Ab-HRP (511203) were purchased from ZENBIO. Goat anti-rabbit Ab-AF594 (SA00013-4), rabbit anti-phospho-AKT Ab (66444-1-lg), and mouse anti-GAPDH Ab (60004-1-Ig) were purchased from Proteintech. Mouse anti-GFP Ab (M20004) was purchased from Abmart. Rabbit anti-LPAR6 Ab was purchased from Immunoway. Rabbit anti-TWIST1 Ab (07769), anti-PI3K Ab (14915), anti-phospho-PI3K Ab (14485), anti-ITGA5 Ab (17098), anti-TSPAN4 Ab (18433) were purchased from UpingBio. Rabbit anti-MMP2 Ab (SRP13502) was purchased from Saier Biotech. Mouse anti-CD147 Ab (H18) was originally produced in our lab [1-3]. Biotin-labeled mouse anti-CD147 Ab (E-AB-F1056B) was purchased from Elabscience.

Streptavidin beads (PuriMag^TM^G) were purchased from PuriMag Biotech. Sorafenib (S45467) was purchased from Yuanye Biotechnology. Doxycycline hydrochloride (D302150) and polyphyllin I (P424367) were purchased from Aladdin. Wortmannin (681675) and Genistein (G810424) were purchased from MACKLIN. EIPA (E10484) was purchased from SAITONG. Chlorpromazine (CPZ, HY-12708) was purchased from MCE. FITC-Dextran (61224ES50) was purchased from YEASEN. FITC-CTxB (C1655) and FITC-Tfn (40-009-090-050) were purchased from SIGMA. Lipofectamine 2000 (Lipo2K, GK20005) was purchased from Good Laboratory Practice Bioscience. Fibronectin (F8180) and CCK8 Kit (CA1210) were purchased from Solarbio. Annexin V/7-AAD apoptosis Kit (A5001-02A-L) was purchased from SIMUBIOTECH. Periodic Acid-Schiff Staining Kit (C0142S) was purchased from Beyotime. An immunohistochemical kit (PV-9000) was purchased from ZSGB-BIO. 100-kDa ultrafiltration tubes (UFC9100) were purchased from Merck Millipore Billerica. The TTCPy molecular probe [4] was supplied by Dr. Jie Cui (Shenzhen University).

**2. Knock-down of the target gene.**

Three LPAR6-targeting siRNAs (LPAR6-KD1: GGUUAAUAUCCAAUUGUGU(dT)(dT). LPAR6-KD2: GUUCUUAACCUGUAUUAGU(dT)(dT). LPAR6-KD3: CCUGUUACAUUAAGUAGAA(dT)(dT)) were generated by Tsingke biotech. Lipo2K-based transfection of LPAR6 siRNA duplexes into MHCC-97H cells and HCCLM3 cells was performed as previously described [2, 5-9]. The knockdown effect was confirmed by western blot using primary Ab followed by corresponding secondary HRP-Ab.

**3.** **Isolation of EVs, migrasomes and migrasome-derived nanoparticles (MDNPs).**

The Isolation of EVs was performed as our previous study [5] (Supplementary Fig S13A). The ultrafiltration method was employed to isolate migrasomes [10, 11]. In brief, HCC cells at 40-50% confluence were washed twice with phosphate-buffered saline (PBS), digested, harvested, and subjected to centrifugation at 1000 g for 10 minutes, followed by centrifugation at 4000 g for 20 minutes to remove cells and large debris. The supernatants were then collected and retained. Migrasomes and MDNPs were separated according to specific operational protocols. For migrasome isolation, the collected supernatant was filtered through a 0.45 μm filter, and the filter was inverted and compressed using a medical syringe containing 5 mL of PBS. The resulting filtrate was concentrated using a 100 kDa ultrafiltration tube at 2000 g for 5 minutes at 4°C to collect the pellet, which constituted the migrasomes. For the isolation of MDNPs, the collected supernatant was filtered through a 0.45 μm filter, transferred to new tubes, and subjected to ultracentrifugation at 17,000 g for 30 minutes at 4°C to pellet the MDNPs. The supernatant was immediately discarded, and the precipitate was washed with precooled PBS and subjected to further centrifugation at 17,000 g for 30 minutes at 4°C. Finally, the pellets were gathered and resuspended with PBS and concentrated through a 100-kDa ultrafiltration tube (Merck Millipore, Billerica, MA, USA) at 2000 g for 5 min at 4 °C (Supplementary Fig S13B).

To isolate CD147⁺ migrasomes, migrasomes were first extracted as described above. Streptavidin-coated magnetic beads (15 μg) were incubated with biotin-labeled anti-CD147 antibody (15 μg) overnight at 4 ℃. After separation on a magnetic stand, the antibody-conjugated beads were collected and incubated with migrasomes for 16 hours at 4 ℃. Following magnetic separation, the supernatant contained CD147^-^ migrasomes, while CD147⁺ migrasomes were recovered from the antibody-conjugated beads by washing (Supplementary Fig S13C).

To isolate migrasomes from serum samples, blood samples of HCC patients with/without sorafenib treatment were collected in pro-coagulation vacuum tubes using standard venipuncture protocols. Large debris was removed by centrifugation at 1000 g for 10 min followed by 4000 g for 20 min. Crude migrasomes were collected by centrifugation at 20,000 g for 30 min, then resuspended with PBS [12].

**4. Identification of migrasomes.**

The morphology of isolated migrasomes was checked by transmission electron microscopy (TEM) as the same methods previously described [5, 6, 9, 13]. Briefly, suspended migrasome suspensions were mounted on copper grids for 5 minutes. Excess fluid was discarded, and migrasome particles were stained with phosphotungstic acid for two minutes. The remaining staining solution was removed, and the grids were washed with ddH_2_O, followed by air-drying. Migrasome samples were imaged by JEM-1400 TEM at a voltage of 120 kV.

The size distribution of isolated migrasomes was analyzed by the particle tracking analyzer (Metrix Zetaview nanoparticle). Data was collected over 2 minutes for each sample.

The protein concentration of the migrasomes and MDNPs’ lysate was determined by the standard BCA protein kit. The expression of migrasomes-specific markers (ITGA5, TSPAN4 and EOGT) was further determined by Western blotting analysis.

**5. Cellular viability assay.**

HCC cells were seeded at a density of 1x10^4^/ml in 96-well plates and grown for 24 h. Sorafenib at IC50 (6 μg/mL for MHCC-97H and 8 μg/mL for HCCLM3) was added for 36 hours, with or without migrasome co-culture (2 μg/well) for 24 hours. Enhanced CCK8 solution was further incubated with cells for 1 h at 37 ℃. Absorbance was monitored by a microplate reader (450 nm), and cell viability was calculated.

**6. Cellular apoptosis assay.**

Cell apoptosis s was measured by the Annexin V/7-AAD apoptosis detection kit according to the manufacturer’s instructions. Briefly, HCC cells were pretreated with sorafenib as described above or cocultured with migrasomes (2 μg/well) for 24 hours. Cells were carefully harvested, washed twice with PBS, and stained with Annexin V and 7-AAD diluent for 20 minutes and subjected to analysis using flow cytometry. Data were recorded from 1 × 10^4^ cells in triplicate per condition.

**7. Migrasome imaging in HCC cells by HIS-SIM.**

Migrasome imaging was measured by high intelligent and sensitive structured illumination microscopy (HIS-SIM, CSR Biotech, Guangzhou) as previously described [4]. Lipo2K-based transient transfection of FUGW-CAG-TSPAN4-GFP plasmid into MHCC-97H cells and HCCLM3 cells was performed and constructed TSPAN4-GFP overexpression cell lines. TTCPy simultaneously labeled migrasomes in TSPAN4-GFP overexpression cell lines. TTCPy staining solution was prepared at a concentration of 2 μg/mL (0.1% DMSO) for migrasome labeling. Cells were seeded in 35 mm glass bottom dishes (Cellvis, D35-20-1.5-N) pre-coated with fibronectin (10 μg/mL) and incubated for 8 h, then washed with PBS and fixed using 2.5% glutaraldehyde for 20 minutes. Subsequently, cells were stained with TTCPy (2 μg/mL, 15min) for fluorescent imaging. The fixed cell super-resolution imaging was performed on HIS-SIM using a ×100 oil immersion lens, and sparse deconvolution was employed for image reconstruction to improve the imaging resolution and contrast.

**8. Migrasome-labeling, uptake and colocalization analysis.**

Lipo2K-based transfection of pCMV-LPAR6(human)-3×FLAG-Neo plasmid into MHCC-97H cells was performed and constructed LPAR6 overexpression cell lines. Migrasome samples were mixed with TTCPy probe solution in the dark for 30 min. Unlabeled dyes were removed by centrifuging the mixture at 20000 g for 30 min and labeled migrasome pellet (TTCPy-Migs) was resuspended in PBS.

To determine the migrasome-uptake pathway, FITC-Dextran (70 kDa, macropinocytosis maker, 4 mg/mL), FITC-Tfn (CME marker, 25 μg/mL) and FITC-CTxB (CDE marker, 10 μg/mL) were respectively co-incubated with TTCPy-Migrasomes (20 μg/mL) in HCC cells at 37 °C for 1h.

Migrasome uptake measured by confocal imaging was the similar approach as EV uptake [5, 14, 15]. HCC cells growing in chamber slides were first starved with serum-free medium for 16 h, then incubated with TTCPy-Migrasomes (20 μg/mL) at 37 °C for 1 hour. To inhibit the uptake of TMR-dextran or TTCPy-Migrasomes, HCC cells were pretreated with different concentrations of EIPA (50 μM), wortmannin (0.5 μM), genistein (200 μM), and CPZ (10 μM) for 4 hours. After quick-rinsing, nuclei-staining with DAPI and migrasome uptake imaging by confocal microscope (Olympus FV 1000). Total particle per cell area was evaluated from at least 8 fields. The images were processed by ImageJ software, and the co-localization coefficient was calculated.

**9. Scanning electron microscope (SEM).**

HCC cells were inoculated onto glass slides in 24-well plates at a density of 40%, cultured for 16 h, and fixed overnight with 3% glutaraldehyde at 4 °C. Rinsed the glass slides for 15 min in 0.1M PBS and dehydrated the glass slides using an ethanol gradient (30%-50%-70%-80%-90%-100%). Lastly, the migrasomes of HCC cells were observed via SEM (JSM-7900F, JEOL, Japan).

**10. Western blotting.**

HCC cells were lysed in RIPA buffer. The protein samples were quantified by a BCA kit, resolved by SDS-PAGE, transferred onto PVDF membranes, blotted respectively with primary Abs as following (mouse anti-CD147 Ab, anti-AKT Ab, 1:2000; anti-GFP Ab, 1:1000; anti-GAPDH Ab, 1:5000; Rabbit anti-HIF1𝛼 Ab, 1:1000; anti-EOGT Ab, 1:800; anti-VE-cadherin Ab, 1:800; anti-E-cadherin Ab (1:1000), anti-CD63 Ab, 1:1000; anti-TSG101 Ab, 1:1000; anti-Calnexin Ab, 1:1000; anti-p-AKT Ab, 1:1000; anti-TWIST1 Ab, 1:1000; anti-PI3K Ab, 1:1000; anti-p-PI3K Ab 1:1000; anti-ITGA5 Ab 1:1000; anti-TSPAN4 Ab, 1:1000), and finally detected with corresponding HRP-labeled secondary Abs (goat anti-mouse Ab, 1:10000; goat anti-rabbit Ab, 1:10000) via Tanon 5200 automatic chemiluminescence imaging analysis system (Shanghai, China). The relative protein expression was calculated as the ratio of gray value of protein to be tested to that of GAPDH.

**11. HCC-xenografted mouse models.**

HCC cell-derived xenograft (CDX) mouse models were further created as previously described [3, 5, 16]. Briefly, HCC cells (1 × 10^7^) were subcutaneously injected into the armpits of NOD/SCID mice. Tumor growth was monitored once every two days by measuring the diameter of tumors with a caliper, and tumor volumes were calculated according to the formula: Volume = (D × d^2^/2), where D is the longest axis of the tumor and d is the shortest length of a prolate ellipse. When the tumor volume reached approximately 0.4 cm in diameter, all mice were randomly grouped (n=5/group) and administered with sorafenib (40 mg kg^−1^, i.g., qod, 21 days) intragastrically every two days. Migrasomes (0.5 mg kg^−1^, i.p., qod, 21 days), Doxycycline (5 mg kg^−1^, i.p., q3d, 21 days), Polyphyllin I (2 mg kg^−1^, i.p., q3d, 21 days), and Wortmannin (1 mg kg^−1^, i.p., q3d, 21 days) were intraperitoneally administered twice a week. Tumor volume was then continuously monitored for 23 days, and all the mice were euthanized at experiment endpoint. The xenografts were preserved in 4% PFA solution for further study.

**12. Immunohistochemical staining and PAS/CD31 staining.**

Peripheral bloods of 14 HCC patients (with/without sorafenib treatment) and 14 healthy individual were collected from the Tianjin Medical University Cancer Institute & Hospital and Tianjin First Central Hospital (China). Fresh HCC tissues and paracancerous tissues (PNT) were collected from HCC patients with hepatectomy and had not undergone radiotherapy before the operation. The collected HCC tissues were staged according to the TNM classification system, in which T indicates the size and extent of the primary tumor, N denotes regional lymph node involvement, and M represents the presence of distant metastasis. Based on the combination of these three parameters, tumors were classified into stages I through III, with higher stages indicating more advanced disease severity. Each tissue block was sliced (diameter: 0.6 mm), followed by fixation with 4% paraformaldehyde (for IHC and PAS/CD31 staining). Slices of embedded tissues were mounted on glass slices after being sliced into 6 𝜇m sections.

Immunohistological analysis was performed as described previously [2, 3, 5, 16, 17]. Briefly, after deparaffinization, rehydration, and antigen-retrieval treatment with Tris-EDTA buffer (pH 9.0) at 121 °C for 5 min, slides were treated with hydrogen peroxide to quench endogenous peroxidase, blocked with goat serum, and incubated with primary antibodies (rabbit anti-TWIST1 Ab, 1:100; anti-VE-cadherin Ab, 1:100; anti-E-cadherin Ab, 1:100; anti-MMP2 Ab, 1:100; anti-MMP9 Ab, 1:100; anti-VEGFR Ab, 1:100) overnight at 4 °C. Non-immune rabbit IgG was used as a negative control. IHC staining was performed with the EnvisionTM two-step system (Dako, USA). Slides were treated with 3, 3-diaminobenzidine and counterstained with hematoxylin. Immunopositivity was independently assessed and scored by two pathologists, who were blinded to the clinical data.

For PAS/CD31 double staining, the procedure was performed as previous described [18, 19]. Sections were stained with CD31 primary antibody (rabbit anti-CD31 Ab, 1:100) and then treated with 0.5% periodic acid solution for 8 min and rinsed with distilled water for 5 min. In a dark chamber, sections were treated with Schiff solution for 30 min at 37℃. After rinsing with distilled water, sections were counterstained with hematoxylin and dehydrated.

**13.** **Bioinformatics analysis.**

Bioinformatics analyses were performed similarly as described previously [2, 5-7, 17]. The Human Protein Atlas (HPA, http://www.proteinatlas.org/) database was employed to analyze the tissue expression level of HIF1𝛼 and ITGA5. GEO (<https://www.ncbi.nlm>..nih.gov/gds) database was employed to analyze the mRNA transcriptional level of HIF1𝛼 and TSPAN4 in HCC tissues (GSE109211) and cell lines (GSE128686). We enlarged the training dataset in equal proportions. Expression-upregulated genes in SFR-HCC cells relative to SFS-cells were identified with absolute fold change >2 and adjusted Padj value <0.05. Survival analysis of HIF1𝛼, ITGA5 and LPAR6 in HCC patients treated with sorafenib (n=29) was conducted using TCGA database (<https://portal.gdc.cancer.gov/>). The analysis of gene co-expression between HIF1𝛼 and ITGA5, EOGT, TSPAN4 was also employed by the TCGA database.

**14. Transcriptome sequencing (RNA-seq)**

Treat MHCC-97H cells with PBS, migrasomes from MHCC-97H cells cultured in normoxia (2 𝜇g, 24h, group Normo-Migs), migrasomes from MHCC-97H cells cultured in hypoxia (2 𝜇g, 24h, group Hypo-Migs) or migrasomes from CD147 knockout MHCC-97H cells cultured in hypoxia (2 𝜇g, 24h, group CD147KO-Migs). Extract total RNA from these cells. mRNA was purified from total RNA using poly-T oligo-attached magnetic beads. After fragmentation, the first strand cDNA was synthesized using random hexamer primers followed by the second strand cDNA synthesis. The library was ready after end repair, A-tailing, adapter ligation, size selection, amplification, and purification.

For the differential expression gene (DEG) analysis, DESeq2 provides statistical programs for determining differential expression in digital gene expression data using models based on negative binomial distribution. The resulting P-value is adjusted using the Benjamini and Hochberg’s methods to control the error discovery rate. Padj <= 0.05 and |log2(foldchange)| >= 1 were used as the screening criteria for DEGs.

The gene set enrichment analysis (GSEA) mainly included three steps: ① According to the expression data of all genes, calculate the difference between groups of each gene (signal2 noise), and then form a well-ordered list of genes. ② Calculate the Enrichment Score (ES) of the gene set. ③ Calculate the significance level of ES.

The KEGG enrichment analysis was implemented by cluster Profiler R package. The corrected P-value less than 0.05 were considered significantly enriched. The results were visualized using the online tool (https://magic-plus.novogene.com/#/project/list).

**15. Statistical analysis**

All the results are presented as mean ± SEM values. Statistical analyses were executed with GraphPad Prism (V8.3.0) by using Student’s *t* test. Significant difference was defined as * *P* < 0.05, ** *P* < 0.01, and ns: no significant difference. The detailed statistical analysis and sample size applied to each experiment was presented in the corresponding figure legends.

**16. Study approval**

Animal experiments were approved by the Nankai University Animal Care and Use Committee (2021-SYDWLL-000479). Human blood samples were obtained from health examiners and HCC patients, and surgical specimens were obtained from HCC patients (informed consent was obtained from the Review Board of Nankai University, NKUIRB2021043).

**REFERENCES**

1.	Zhao P, Zhang W, Wang SJ, Yu XL, Tang J, Huang W, et al. HAb18G/CD147 promotes cell motility by regulating annexin II-activated RhoA and Rac1 signaling pathways in hepatocellular carcinoma cells. Hepatology. 2011;54(6):2012-24.

2.	Qi S, Su L, Li J, Zhao P, Zhang Q, Niu X, et al. YIPF2 is a novel Rab-GDF that enhances HCC malignant phenotypes by facilitating CD147 endocytic recycle. Cell Death Dis. 2019;10(6):462.

3.	Qi FZ, Su HS, Wang B, Qian LM, Wang Y, Wang CH, et al. Hypoxia-activated ADCC-enhanced humanized anti-CD147 antibody for liver cancer imaging and targeted therapy with improved selectivity. MedComm (2020). 2024;5(3):e512.

4.	Cui J, Zhang F, Jiang D, Liu B, Zhang H, Niu N, et al. An AIE-active near-infrared molecular probe for migrasome labeling. Biomaterials. 2025;319:123213.

5.	Qian L, Fu Z, Chen P, Guo Y, Li Y, Wang Y, et al. Lysophosphatidic acid-induced Arf6-driven macropinocytosis of CD147(+) extracellular vesicles promotes sorafenib resistance of hepatocellular carcinoma. Int J Biol Sci. 2026;22(1):220-38.

6.	Su L, Sun Z, Qi F, Su H, Qian L, Li J, et al. GRP75-driven, cell-cycle-dependent macropinocytosis of Tat/pDNA-Ca(2+) nanoparticles underlies distinct gene therapy effect in ovarian cancer. J Nanobiotechnology. 2022;20(1):340.

7.	Li J, Qi F, Su H, Zhang C, Zhang Q, Chen Y, et al. GRP75-faciliated Mitochondria-associated ER Membrane (MAM) Integrity controls Cisplatin-resistance in Ovarian Cancer Patients. Int J Biol Sci. 2022;18(7):2914-31.

8.	Niu X, Su L, Qi S, Gao Z, Zhang Q, Zhang S. GRP75 modulates oncogenic Dbl-driven endocytosis derailed via the CHIP-mediated ubiquitin degradation pathway. Cell Death Dis. 2018;9(10):971.

9.	Niu X, Gao Z, Qi S, Su L, Yang N, Luan X, et al. Macropinocytosis activated by oncogenic Dbl enables specific targeted delivery of Tat/pDNA nano-complexes into ovarian cancer cells. Int J Nanomedicine. 2018;13:4895-911.

10.	Ma Y, Li T, Zhao L, Zhou D, Dong L, Xu Z, et al. Isolation and characterization of extracellular vesicle-like nanoparticles derived from migrasomes. Febs j. 2023;290(13):3359-68.

11.	Liu W, Li L, Bai X, Zhang M, Lv W, Ma Y, et al. Osteosarcoma Cell-Derived Migrasomes Promote Macrophage M2 Polarization to Aggravate Osteosarcoma Proliferation and Metastasis. Adv Sci (Weinh). 2025;12(17):e2409870.

12.	Zhao X, Lei Y, Zheng J, Peng J, Li Y, Yu L, et al. Identification of markers for migrasome detection. Cell Discov. 2019;5:27.

13.	Song N, Sun Z, Wang B, Liu X, Hu B, Chen N, et al. Suicide gene delivery by morphology-adaptable enantiomeric peptide assemblies for combined ovarian cancer therapy. Acta Biomater. 2024;175:250-61.

14.	Costa Verdera H, Gitz-Francois JJ, Schiffelers RM, Vader P. Cellular uptake of extracellular vesicles is mediated by clathrin-independent endocytosis and macropinocytosis. J Control Release. 2017;266:100-8.

15.	Wang X, Qiao D, Chen L, Xu M, Chen S, Huang L, et al. Chemotherapeutic drugs stimulate the release and recycling of extracellular vesicles to assist cancer cells in developing an urgent chemoresistance. Mol Cancer. 2019;18(1):182.

16.	Wang B, Qi FZ, Chen P, Qian LM, Su HS, Wang Y, et al. Hypoxia-activated selectivity-improved anti-PKM2 antibody combined with prodrug TH-302 for potentiated targeting therapy in hepatocellular carcinoma. Int J Biol Sci. 2024;20(5):1634-51.

17.	Qi S, Su L, Li J, Zhang C, Ma Z, Liu G, et al. Arf6-driven endocytic recycling of CD147 determines HCC malignant phenotypes. J Exp Clin Cancer Res. 2019;38(1):471.

18.	Xia T, Yin H, Zhu Q, Zhang K, Xie H, Shan Y, et al. DDX5 super-enhancer promotes vasculogenic mimicry formation and metastasis in nasopharyngeal carcinoma by enhancing ADAM10 transcription. Cell Rep Med. 2025;6(6):102146.

19.	Wu S, Yao C, Fang L, Sun Y, Zhao M, Chen J, et al. ATPase Inhibitory Factor 1 Drives Mitochondrial Energy Metabolic Reprogramming to Promote HCC Vasculogenic Mimicry via the ESR1/miR-20a-3p/GNAZ Pathway. Research (Wash D C). 2025;8:0998.
