# Supplementary material for: CD147-positive migrasome macropinocytosis promotes HCC sorafenib resistance via inducing vasculogenic mimicry triggered by PI3K/AKT/TWIST1 signaling
Source: Cell Death Dis. 2026 Jun 15;17(1):750. doi: 10.1038/s41419-026-08972-y (PMC13500725; doi:10.1038/s41419-026-08972-y)
Supplement: Supplementary file 3 [file 41419_2026_8972_MOESM3_ESM.docx]

Figure 2

Figure 4

Figure 5

Figure S1

Figure S2
